# Sharp $N^{3/4}$ Law for the Minimizers of the Edge-Isoperimetric Problem on the Triangular Lattice

**DOI:** 10.1007/s00332-016-9346-1

**Published:** 2016-11-05

**Authors:** Elisa Davoli, Paolo Piovano, Ulisse Stefanelli

**Affiliations:** 1 Faculty of Mathematics, University of Vienna, Oskar-Morgenstern-Platz 1, 1090 Vienna, Austria; 2 Istituto di Matematica Applicata e Tecnologie Informatiche “E. Magenes” - CNR, v. Ferrata 1, 27100 Pavia, Italy

**Keywords:** Edge-isoperimetric problem, Edge perimeter, Triangular lattice, Isoperimetric inequality, Wulff shape, *N*^3/4^ law, 82D25

## Abstract

We investigate the edge-isoperimetric problem (EIP) for sets of *n* points in the triangular lattice by emphasizing its relation with the emergence of the Wulff shape in the crystallization problem. By introducing a suitable notion of perimeter and area, EIP minimizers are characterized as extremizers of an isoperimetric inequality: they attain maximal area and minimal perimeter among connected configurations. The maximal area and minimal perimeter are explicitly quantified in terms of *n*. In view of this isoperimetric characterizations, EIP minimizers $M_n$ are seen to be given by hexagonal configurations with some extra points at their boundary. By a careful computation of the cardinality of these extra points, minimizers $M_n$ are estimated to deviate from such hexagonal configurations by at most $K_t\, n^{3/4}+\mathrm{o}(n^{3/4})$ points. The constant $K_t$ is explicitly determined and shown to be sharp.

## Introduction

This paper is concerned with the *edge-isoperimetric problem* (EIP) in the triangular lattice

$$\begin{aligned} {\mathcal {L}}_t:=\{m {\varvec{t}}_1+n {\varvec{t}}_2\,:\,m,n\in \mathbb {Z}\}\quad \text { for } {\varvec{t}}_1:=\left( 1,0\right) \text { and } {\varvec{t}}_2:=\left( \frac{1}{2},\frac{\sqrt{3}}{2}\right) . \end{aligned}$$

Let ${\mathcal {C}}_n$ be the family of sets $C_n$ containing *n* distinct elements $x_1,\ldots , x_n$ in ${\mathcal {L}}_t$. The *edge perimeter* $|\Theta (C_n)|$ of a set $C_n\in {\mathcal {C}}_n$ is the cardinality of the *edge boundary* $\Theta$ of $C_n$ defined by

1 $$\begin{aligned} \Theta (C_n):=\{ (x_i,x_j) \ : \ |x_i-x_j|=1, x_i\in C_n \text { and } x_j\in {\mathcal {L}}_t\setminus C_n \}. \end{aligned}$$

Note that, with a slight abuse of notation, the symbol $|\cdot |$ denotes, according to the context, both the cardinality of a set and the euclidean norm in $\mathbb {R}^2$. The EIP over the family ${\mathcal {C}}_n$ consists in characterizing the solutions to the minimum problem:

2 $$\begin{aligned} \theta _n:=\min _{C_n\in {\mathcal {C}}_n} |\Theta (C_n)|. \end{aligned}$$

Our main aim is to provide a characterization of the minimizers $M_n$ of (2) as extremizers of a suitable isoperimetric inequality (see Theorem 1.1) and to show that there exists a *hexagonal Wulff shape* in ${\mathcal {L}}_t$ from which $M_n$ differs by at most

3 $$\begin{aligned} K_t\, n^{3/4}+\mathrm{o}(n^{3/4}) \end{aligned}$$

points (see Theorem 1.2). A crucial issue of our analysis is that both the exponent and the constant in front of the leading term in (3) are explicitly determined and optimal (see Theorem 1.4).

The EIP is a classical combinatorial problem. We refer to Bezrukov (1999), Harper (2004) for the description of this problem in various settings and for a review of the corresponding results available in the literature. The importance of the EIP is however not only theoretical, since the edge perimeter (and similar notions) bears relevance in problems from *machine learning*, such as classification and clustering (see Trillos and Slepcev 2016 and references therein). Note, however, that in this other more statistical setting the edge perimeter is not defined for configurations contained in a specific lattice, but for point clouds obtained as random samples.

We shall emphasize the link between the EIP and the *Crystallization Problem* (CP). For this reason, we will often refer to the sets $C_n\in {\mathcal {C}}_n$ as *configurations of particles* in ${\mathcal {L}}_t$ and to minimal configurations as *ground states*. The CP consists in analytically explaining why particles at low temperature arrange in periodic lattices by proving that the minima of a suitable *configurational energy* are subsets of a regular lattice. At low temperatures, particle interactions are expected to be essentially determined by particle positions. In this classical setting, all available CP results in the literature with respect to a finite number *n* of particles are in two dimensions for a phenomenological energy *E* defined from ${\mathbb {R}}^{2n}$, the set of possible particle positions, to ${\mathbb {R}}\cup \{+\infty \}$. In Heitmann and Radin (1980), Radin (1981) the energy *E* takes the form

4 $$\begin{aligned} E(\{y_1,\ldots ,y_n\}):= \frac{1}{2}\sum _{i\ne j}v_2(|y_i-y_j|) \end{aligned}$$

for specific potentials $v_2:[0,\infty )\rightarrow \mathbb {R}\cup \{+\infty \}$ representing two-body interactions. Additional three-body interaction terms have been included in the energy in Mainini and Stefanelli (2014), Mainini et al. (2014a, b). We also refer the reader to E and Li (2009), Flatley and Theil (2015), Theil (2006) for results in the *thermodynamic limit* with a Lennard-Jones-like potential $v_2$ not vanishing at a certain distance and to Blanc and Lewin (2015) for a general review on the CP.

The link between the EIP on ${\mathcal {L}}_t$ and the CP resides on the fact that when only two-body and short-ranged interactions are considered, the minima of *E* are expected to be subsets of a triangular lattice. The fact that ground states are subsets of ${\mathcal {L}}_t$ has been analytically shown in Heitmann and Radin (1980) and Radin (1981), respectively, with $v_2:=v_\mathrm{sticky}$, where $v_\mathrm{sticky}$ is the *sticky-disk* potential, i.e.,

5 $$\begin{aligned} v_\mathrm{sticky}(\ell ):={\left\{ \begin{array}{ll} +\infty \quad &{}\text {if } \ell \in [0,1)\\ -1\quad &{}\text {if } \ell =1\\ 0\quad &{}\text {if } \ell >1\,, \end{array}\right. } \end{aligned}$$

and $v_2:=v_\mathrm{soft}$, where $v_\mathrm{soft}$ is the *soft-disk* potential, i.e.,

6 $$\begin{aligned} v_\mathrm{soft}(\ell ):={\left\{ \begin{array}{ll} +\infty \quad &{}\text {if }\ell \in [0,1)\\ 24\ell -25\quad &{}\text {if }\ell \in [1,25/24]\\ 0\quad &{}\text {if }\ell >25/24\,. \end{array}\right. } \end{aligned}$$

In particular with both the choices (5) and (6) for $v_2$, we have that

7 $$\begin{aligned} E(C_n)=-|B(C_n)| \end{aligned}$$

for every $C_n\in {\mathcal {C}}_n$. Here, the set

8 $$\begin{aligned} B(C_n):=\{ (x_i,x_j) \ : \ |x_i-x_j|=1, i<j, \text { and } x_i, x_j\in C_n\} \end{aligned}$$

represents the *bonds* of $C_n\in {\mathcal {C}}_n$. Note that the definition of $B(C_n)$ in (8) is independent of the order in which the elements of $C_n$ are labeled. The number of bonds of $C_n$ with an endpoint in $x_i$ will be instead denoted by

9 $$\begin{aligned} b(x_i)=|\{j\in \{1,\ldots ,n\} \ : \ (x_i,x_j)\in B(C_n) \text { or } (x_j,x_i)\in B(C_n) \}| \end{aligned}$$

for every $x_i\in C_n$. The link between the EIP and the CP consists in the fact that by (1), (7), and (9) we have that

10 $$\begin{aligned} |\Theta (C_n)|&=\sum _{i=1}^n\left( 6-b(x_i)\right) =6n-\sum _{i=1}^n b(x_i)\nonumber \\&= 6n \,-\, 2 |B(C_n)|= 6n\,+\,2E(C_n) \end{aligned}$$

for every $C_n\in {\mathcal {C}}_n$, since the degree of ${\mathcal {L}}_t$ is 6.

In view of (10) minimizing *E* among configurations in ${\mathcal {C}}_n$ is equivalent to the EIP (2), and since for both the choices (5) and (6) for $v_2$ by Heitmann and Radin (1980), Radin (1981) ground states belong to ${\mathcal {C}}_n$, the ground states of the CP correspond to the minimizers of the EIP. Furthermore, in Heitmann and Radin (1980), Radin (1981) the energy of ground states with *n* particles has been also explicitly quantified in terms of *n* to be equal to

11 $$\begin{aligned} e_n:=-\lfloor 3n-\sqrt{12n-3}\rfloor =-3n+\lceil \sqrt{12n-3}\rceil \end{aligned}$$

where $\lfloor x\rfloor :=\max \{z\in {\mathbb {Z}}:z\le x\}$ and $\lceil x\rceil :=\min \{z\in {\mathbb {Z}}:x\le z\}$ denote the standard right- and left-continuous functions, respectively. Therefore, (10) and (11) entails also a characterization of $\theta _n$ in terms of *n*, i.e.,

12 $$\begin{aligned} \theta _n= 6n +2 e_n=2\lceil \sqrt{12n-3}\rceil . \end{aligned}$$

A first property of the minimizers of (2) has been provided in Harper (2004), Theorem 7.2 where it is shown that the EIP has the *nested-solution property*, i.e., there exists a total order $\tau :\mathbb {N}\rightarrow {\mathcal {L}}_t$ such that for all $n\in \mathbb {N}$ the configuration

$$\begin{aligned} D_n:=\{x_{\tau (1)},\ldots ,x_{\tau (n)}\} \end{aligned}$$

is a solution of (2) (see Proposition 2.1 and the discussion below for the definition of $\tau$). Given the symmetry of the configurations $D_n$, we will refer to them as *daisies* in the following. Since solutions of the EIP are in general nonunique, the aim of this paper is to characterize them all.

In this paper, we provide a first characterization of the minimizers $M_n$ of the EIP by introducing an isoperimetric inequality in terms of suitable notions of *area* and *perimeter* of configurations in ${\mathcal {C}}_n$ and by showing that the connected minimizers $M_n$ of the EIP are optimal with respect to it. We refer here the reader to (25) and (26) for the definition of the area $A(C_n)$ and the perimeter $P(C_n)$ of a configuration $C_n\in {\mathcal {C}}_n$. Note also that we say that a configuration $C_n$ is connected if given any two points $x_i,x_j\in C_n$ then there exists a sequence $y_k$ of points in $C_n$ with $k=1,\ldots ,K$ for some $K\in \mathbb {N}$ such that $y_1=x_i$, $y_K=x_j$, and either $(y_k,y_{k+1})$ or $(y_{k+1},y_{k})$ is in $B(C_n)$ for every $k=1,\ldots , K-1$. It easily follows that minimizers of the EIP need to be connected. Our isoperimetric characterization reads as follows.

### Theorem 1.1

(Isoperimetric characterization) Every connected configuration $C_n\in {\mathcal {C}}_n$ satisfies

13 $$\begin{aligned} \sqrt{A(C_n)} \le k_n P(C_n), \end{aligned}$$

where

14 $$\begin{aligned} k_n := \frac{\sqrt{-2\theta _n + 8n +4 }}{\theta _n -6}\,. \end{aligned}$$

Moreover, connected minimizers $M_n\in {\mathcal {C}}_n$ of the EIP correspond to those configurations for which (13) holds with the equality. Finally, connected minimizers attain the maximal area $a_n:=-\theta _n/2+ 2n +1$ and the minimal perimeter $p_n:=\theta _n/2 -3$.

Notice that a similar isoperimetric result has been already achieved in the square lattice in Mainini et al. (2014a) with a different method, based on introducing a rearrangement of the configurations. Theorem 1.1 is instead proved by assigning to each element *x* of a configuration $C_n\in {\mathcal {C}}_n$ a weight $\omega _{C_n}(x)$ that depends on $C_n$ and on the above-mentioned order $\tau$ [see (32)].

Furthermore, we observe that the isoperimetric constant $k_n$ given by (14) satisfies

$$\begin{aligned} k_n\le \frac{1}{\sqrt{6}}\quad \text {for every }n\in \mathbb {N}, \end{aligned}$$

with $k_n=1/\sqrt{6}$ if and only if $n=1+3s+3s^2$ for some $s\in \mathbb {N}$. Note that for $n=1 +3s + 3s^2$, as already observed in Harper (2004), the hexagonal daisy $D_{1 +3s + 3s^2}$ is the unique minimizer of the EIP.

In the following, we will often refer to lattice translations of $D_{1 +3s + 3s^2}$ as *hexagonal configurations with radius* $s\in \mathbb {N}$ since each configuration $D_{1 +3s + 3s^2}$ can be seen as the intersection of ${\mathcal {L}}_t$ and a regular hexagon with side *s*. In order to further characterize the solutions of the EIP, we associate to every minimizer $M_n$ a maximal hexagonal configuration $H_{r_{M_n}}$ that is contained in $M_n$ and we evaluate how much $M_n$ differs from $H_{r_{M_n}}$ (see Sect. 3).

In view of the isoperimetric characterization of the ground states provided by Theorem 1.1, we are able to sharply estimate the *distance* of $M_n$ to $H_{r_{M_n}}$ both in terms of the cardinality of $M_n\setminus H_{r_{M_n}}$ and by making use of empirical measures. We associate to every configuration $C_n=\{x_1,\ldots ,x_n\}$ the empirical measure denoted by $\mu _{C_n}\in M_b({\mathbb {R}}^2)$ (where $M_b({\mathbb {R}}^2)$ is the set of bounded Radon measures in $\mathbb {R}^2$) of the rescaled configuration $\{x_1/\sqrt{n},\ldots ,x_n/\sqrt{n}\}$, i.e.,

$$\begin{aligned} \mu _{C_n}:=\frac{1}{n} \sum _i \delta _{x_i/\sqrt{n}}\,, \end{aligned}$$

and we denote by $\Vert \cdot \Vert$ and $\Vert \cdot \Vert _\mathrm{F}$ the total variation norm and the *flat norm*, respectively (see Whitney 1957 and (72) for the definition of flat norm). Our second main result is the following.

### Theorem 1.2

(Convergence to the Wulff shape). For every sequence of minimizers $M_n$ in ${\mathcal {L}}_t$, there exists a sequence of suitable translations $M^{\prime }_n$ such that

$$\begin{aligned} \mu _{M^{\prime }_n}\rightharpoonup ^* \frac{2}{\sqrt{3}}\chi _W\quad \text {weakly* in the sense of measures}, \end{aligned}$$

where $\chi _{W}$ is the characteristic function of the regular hexagon *W* defined as the convex hull of the vectors

$$\begin{aligned} \left\{ \pm \frac{1}{\sqrt{3}}{\varvec{t}}_1,\,\pm \frac{1}{\sqrt{3}}{\varvec{t}}_2,\,\pm \frac{1}{\sqrt{3}}({\varvec{t}}_2-{\varvec{t}}_1)\right\} . \end{aligned}$$

Furthermore, the following assertions hold true:

15 $$\begin{aligned} \left| M_n\setminus H_{r_{M_n}}\right|\le & {} K_t n^{3/4}+\mathrm{o}(n^{3/4}), \end{aligned}$$

16 $$\begin{aligned} \left\| \mu _{M_n}-\mu _{H_{r_{M_n}}}\right\|\le & {} K_t n^{-1/4}+\mathrm{o}(n^{-1/4}), \end{aligned}$$

17 $$\begin{aligned} \left\| \mu _{M'_n}- \mu _{H_{r_{M_n}}} \right\| _\mathrm{F}\le & {} K_t n^{-1/4}+\mathrm{o}(n^{-1/4}), \end{aligned}$$

and

18 $$\begin{aligned} \left\| \mu _{M'_n}-\frac{2}{\sqrt{3}}\chi _W\right\| _\mathrm{F}\le 2 K_t n^{-1/4}+\mathrm{o}(n^{-1/4}), \end{aligned}$$

where $H_{r_{M_n}}$ is the maximal hexagon associated to $M_n$, and

19 $$\begin{aligned} K_t:=\frac{2}{3^{1/4}}. \end{aligned}$$

The proof of Theorem 1.2 is based on the isoperimetric characterization of the minimizers provided by Theorem 1.1 and relies in a fundamental way on the maximality of the radius $r_{M_n}$ of the maximal hexagonal configuration $H_{r_{M_n}}$. The latter is essential to carefully estimate the number of particles of $M_n$ that reside outside $H_{r_{M_n}}$ in terms of $r_{M_n}$ itself and the minimal perimeter $p_n$. Thanks to this fine estimate we are able to find a lower bound on $r_{M_n}$ in terms of *n* only [see (69)]. In particular, the method provides a lower bound for the radius $r_{M_n}$ that allows us also to estimate from above the discrepancy between the sets $M_n$ and $H_{r_{M_n}}$ in the Hausdorff distance that is defined by

$$\begin{aligned} d_{{\mathcal {H}}}(S_1,S_2)=\max \left\{ \,\displaystyle \sup _{x\in S_1}\,\inf _{y\in S_2}|x-y|,\,\displaystyle \sup _{y\in S_2}\,\displaystyle \inf _{x\in S_1}|x-y|\,\right\} \end{aligned}$$

for nonempty sets $S_1,S_2\subset \mathbb {R}^2$.

### Corollary 1.3

(Hausdorff distance) For any minimizer $M_n$ and its associated maximal hexagon $H_{r_{M_n}}$ there holds

20 $$\begin{aligned} {d_{{\mathcal {H}}}\left( M_n, H_{r_{M_n}}\right) }\le 2\cdot 3^{1/4} n^{1/4} +\mathrm{O}(1). \end{aligned}$$

We observe that in view also of Theorem 1.1 estimates (15) – (18) and (20) provide a measure in different topologies of the fluctuation of the isoperimetric configurations in ${\mathcal {L}}_t$ with respect to corresponding maximal hexagons. Similar estimates have been studied in the context of isoperimetric Borel sets with finite Lebesgue measure in $\mathbb {R}^d$, $d\ge 2$. We refer the reader to Fusco et al. (2008) for the first complete proof of the quantitative isoperimetric inequality in such setting, and to Cicalese and Leonardi (2012), Figalli et al. (2010) for subsequent proofs employing different techniques.

Moreover, Theorem 1.2 appears to be an extension of analogous results obtained in Au Yeung et al. (2012), Schmidt (2013) by using a completely different method hinged on $\Gamma$-convergence. In that context, the set *W* is the asymptotic *Wulff shape* and we will also often refer to *W* in this way. More precisely the minimization problem (4) is reformulated in Au Yeung et al. (2012), Schmidt (2013) in terms of empirical measures by introducing the energy functional

21 $$\begin{aligned} {\mathcal {E}}_n(\mu ):={\left\{ \begin{array}{ll} \displaystyle \int _{\mathbb {R}^2\setminus \mathrm{diag}} \displaystyle \frac{n}{2} v_2(\sqrt{n}|x-y|)\, \mathrm {d}\mu \otimes \mathrm {d}\mu &{}\mu = \mu _{C_n} \text { for some } C_n\in {\mathcal {C}}_n,\\ \infty &{}\text {otherwise} \end{array}\right. }\nonumber \\ \end{aligned}$$

defined on the set of nonnegative Radon measures in $\mathbb {R}^2$ with mass 1, where $v_2$ is (a quantified small perturbation of) the sticky-disk potential (Heitmann and Radin 1980). In Au Yeung et al. (2012), Schmidt (2013) it is proved that the rescaled sequence of functionals $n^{-1/2}(2{\mathcal {E}}_n +6n)$ $\Gamma$-converges with respect to the weak$^*$ convergence of measures to the anisotropic perimeter

22 $$\begin{aligned} {\mathcal {P}}(\mu ):={\left\{ \begin{array}{ll} \displaystyle \int _{\partial ^*S} \varphi (\nu _S)\,\mathrm {d}{\mathcal {H}}^1 &{} \text {if } \mu = \displaystyle \frac{2}{\sqrt{3}}\chi _S \text { for some set } S \text { of finite perimeter}\\ &{}\text {and such that }{\mathcal {L}}^2(S):=\sqrt{3}/2,\\ \infty &{}\text {otherwise} \end{array}\right. } \end{aligned}$$

where $\partial ^*S$ is the reduced boundary of *S*, $\nu _S$ is the outward-pointing normal vector to *S*, ${\mathcal {L}}^2(S)$ is the two-dimensional Lebesgue measure of *S*, ${\mathcal {H}}^1$ is the one-dimensional measure, and the anisotropic density $\varphi$ is defined by

$$\begin{aligned} \varphi (\nu ):=2\left( \nu _2-\frac{\nu _1}{\sqrt{3}}\right) \end{aligned}$$

for every $\nu =(\nu _1, \nu _2)$ with $\nu _1=-\sin \alpha$ and $\nu _2=\cos \alpha$ for $\alpha \in [0,\pi /6]$.

Let us note here that the $\Gamma$-convergence result provided in Au Yeung et al. (2012) can be restated as a $\Gamma$-convergence result for the edge perimeter. In fact, since the energy functional ${\mathcal {E}}_n$ is such that

23 $$\begin{aligned} {\mathcal {E}}_n(\mu _{C_n})=E(C_n) \end{aligned}$$

for every $C_n\in {\mathcal {C}}_n$, by (10) we have that the functional ${\mathcal {T}}_n:={\mathcal {E}}_n(\mu )+6n$ is such that

$$\begin{aligned} {\mathcal {T}}_n(\mu _{C_n})=|\Theta (C_n)| \end{aligned}$$

and $n^{-1/2}{\mathcal {T}}_n$ $\Gamma$-converges with respect to the weak$^*$ convergence of measures to the anisotropic perimeter ${\mathcal {P}}(\mu )$.

Besides the completely independent method, the main achievement of this paper with respect to Au Yeung et al. (2012), Schmidt (2013) is that of sharply estimating the constant $K_t$ in formulas (15), (16), and (17). The deviation of the minimizers from the Wulff shape of order $n^{3/4}$ was exhibited in Schmidt (2013) and referred to as the $n^{3/4}$-*law*. Here we sharpen the result from Schmidt (2013) by determining the optimal constant in estimates (15), (16), and (17). We have the following.

### Theorem 1.4

(Sharpness of the estimates) A sequence of minimizers $M_{n_i}$ satisfying (15) – (17) with equalities can be explicitly constructed for $n_i:=2+3i+3i^2$ with $i\in \mathbb {N}$.

The proof of Theorem 1.4 is based on the estimate:

24 $$\begin{aligned} |M_n\setminus H_{r_{M_n}}|\le K_n n^{3/4}+\mathrm{o}(n^{3/4}) \end{aligned}$$

which holds true for the explicitly determined constant $K_n$ introduced in (73). Estimate (24) is a consequence of the lower bound for the radius $r_{M_n}$ established in the proof of Theorem 1.2, see (69). In fact, a sequence of minimizers ${\bar{M}}_n$ satisfying (24) with equality can be explicitly constructed. Note that such configurations ${\bar{M}}_n$ are singled out among configurations that present extra elements outside their maximal hexagon $H_{{\bar{M}}_n}$ in correspondence of only two consecutive faces of $H_{{\bar{M}}_n}$ (see Fig. 6). Therefore, to establish Theorem 1.4 is enough to show that

$$\begin{aligned} \limsup _{n\rightarrow \infty } K_n =K_t \end{aligned}$$

and to exhibit a subsequence $n_i$ that realizes the limit.

Finally, we notice that our method appears to be implementable in other settings possibly including three-body interactions. This is done for the crystallization problem in the hexagonal lattice ${\mathcal {L}}_h$ in a companion paper (Davoli et al. 2016). Furthermore, we observe that analogous results to Theorem 1.2 were obtained in the context of the crystallization problem in the square lattice in Mainini et al. (2014a, b) with a substantially different method (even though also based on an isoperimetric characterization of the minimizers) resulting only in suboptimal estimates.

The paper is organized as follows. In Sect. 2, we introduce the notions of area *A* and perimeter *P* of configurations $C_n\in {\mathcal {C}}_n$, we define the order $\tau$ in ${\mathcal {L}}_t$, and we introduce the notion of weight $\omega _{C_n}$. Furthermore, in Sect. 2.1 we provide the proof of Theorem 1.1. In Sect. 3, we introduce the notion of maximal hexagons $H_{r_{M_n}}$ associated to minimizers $M_n$ of (2) and we carefully estimate $r_{M_n}$ from below in terms of *n*. In Sect. 4, we use the latter lower bound in order to study the convergence to the Wulff shape by providing the proof of Theorems 1.2 and 1.4 in Sects. 4.1 and 4.2, respectively.

## Isoperimetric Inequality

In this section, we introduce the notion of area and perimeter of a configuration in ${\mathcal {C}}_n$ and we deduce various relations between its area, perimeter, energy and its edge boundary including a isoperimetric inequality.

We define the area *A* of a configuration $C_n\in {\mathcal {C}}_n$ by

25 $$\begin{aligned} A(C_n):=|T(C_n)| \end{aligned}$$

where $T(C_n)$ is the family of ordered triples of elements in $C_n$ forming triangles with unitary edges, i.e.,

$$\begin{aligned} T(C_n):=\{ (x_{i_1},x_{i_2},x_{i_3}): \ x_{i_1},x_{i_2},x_{i_3}\in C_n, i_1<i_2<i_3, \text { and } |x_{i_j}{-}x_{i_k}|=1 \text { for } j\ne k \}. \end{aligned}$$

The definition of $A(C_n)$ is invariant with respect to any relabeling of the particles of $C_n$.

In order to introduce the perimeter of a configuration in ${\mathcal {C}}_n$ let us denote by $F(C_n)\subset \mathbb {R}^2$ the closure of the union of the regions enclosed by the triangles with vertices in $T(C_n)$, and by $G(C_n)\subset \mathbb {R}^2$ the union of all bonds which are not included in $F(C_n)$. The *perimeter* *P* of a regular configuration $C_n\in {\mathcal {C}}_n$ is defined as

26 $$\begin{aligned} P(C_n):={\mathcal {H}}^1(\partial F(C_n))+2{\mathcal {H}}^1(G(C_n))\,, \end{aligned}$$

where ${\mathcal {H}}^1$ is the one-dimensional Hausdorff measure. Note in particular that

$$\begin{aligned} P(C_n)= \lim _{\varepsilon \searrow 0}{\mathcal {H}}^1\Big (\partial \big (\partial F(C_n)\cup G(C_n) + B_\varepsilon \big )\Big ) \end{aligned}$$

where $$B_\varepsilon =\{y \in \mathbb {R}^2 \ : \ |y|\le \varepsilon \}$$.

Since every triangle with vertices in $T(C_n)$ contributes with 3 bonds to $B(C_n)$, by (7) and (25) we have that

27 $$\begin{aligned} 3A(C_n)&= 2\,|B(C_n\cap F(C_n))|\,-\, |B(C_n\cap \partial F(C_n))|\nonumber \\&=-2\,E(C_n\cap F(C_n))-{\mathcal {H}}^1(\partial F(C_n)). \end{aligned}$$

Thus, by recalling (26) and (27) the equality

$$\begin{aligned} {\mathcal {H}}^1(G(C_n))=|B(C_n\cap G(C_n))|=-E(C_n\cap G(C_n)) \end{aligned}$$

yields

$$\begin{aligned} P(C_n)&=-2E(C_n\cap F(C_n))\,-\,3A(C_n) - 2E(C_n\cap G(C_n))\\&=-2E(C_n)\,-\,3A(C_n), \end{aligned}$$

and we conclude that

28 $$\begin{aligned} E(C_n)=-\frac{3}{2}A(C_n)\,-\,\frac{1}{2}P(C_n). \end{aligned}$$

Notice that (28) allows to express the energy of a configuration $C_n$ as a linear combinations of its area and its perimeter, and that by (10) an analogous relation can be deduced for the edge boundary, namely

29 $$\begin{aligned} |\Theta (C_n)|= 6n-3A(C_n)-P(C_n). \end{aligned}$$

As already discussed in the introduction, in view of (10) we are able to combine the exact quantification of the ground-state energy *E* established in Heitmann and Radin (1980), Radin (1981) with the nested-solution property provided by Harper (2004), Theorem 7.2. We record this fact in the following result that we state here without proof.

### Proposition 2.1

There exists a total order $\tau :\mathbb {N}\rightarrow {\mathcal {L}}_t$ such that for all $n\in \mathbb {N}$ the configuration $D_n$ defined by $D_n:=\{x_{\tau (1)},\ldots ,x_{\tau (n)}\}$ which we refer to as *daisy* with *n* points is a solution of (2), i.e.,

30 $$\begin{aligned} |\Theta (D_n)|=\min _{C_n\in {\mathcal {L}}_t}|\Theta (C_n)|=\theta _n, \end{aligned}$$

where $\theta _n$ is given by (12).

We remark that the sequence of daisy ground states $\{D_n\}$ satisfies the property that

$$\begin{aligned} D_{n+1}=D_{n}\,\cup \, \{x_{\tau (n+1)}\}. \end{aligned}$$

In particular, within the class of daisy configurations one can pass from a ground state to another by properly adding atoms at the right place, determined by the order $\tau$.

**Fig. 1:**
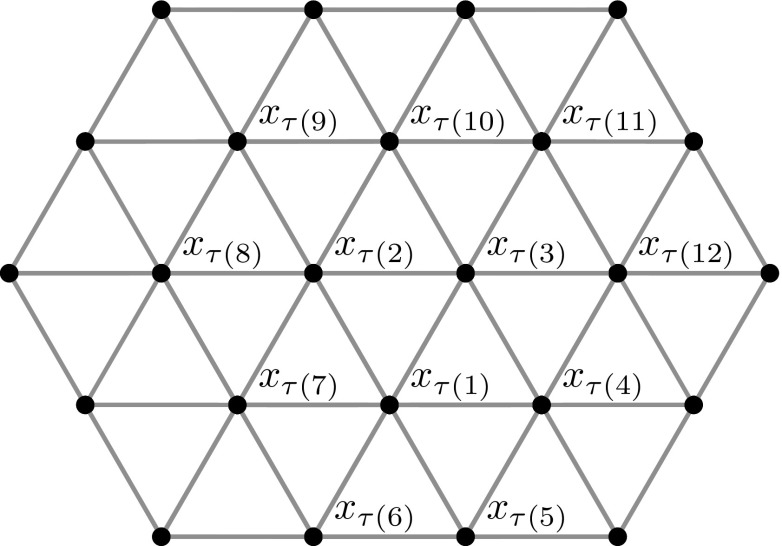
The total order $\tau$ is defined by considering the concentric hexagons centered in $x_{\tau (1)}$ with increasing radii, and by ordering the points clockwise within each hexagon

The total order provided by Theorem 2.1 is not unique. We will consider here the total order $\tau$ on ${\mathcal {L}}_t$ defined by moving clockwise on concentric daisies centered at a fixed point, as the radius of the daisies increases. To be precise, let $x_{\tau (1)}$ be the origin (0, 0) and let $x_{\tau (2)}$ be a point in ${\mathcal {L}}_t$ such that there is an active bond between $x_{\tau (2)}$ and $x_{\tau (1)}$. For $i=3,\ldots ,7,$ we define the points $x_{\tau (i)}\in {\mathcal {L}}_t$ as the vertices of the hexagon $H_k$ with center $x_{\tau (1)}$ and radius 1, numbered clockwise starting from $x_{\tau (2)}$. We then consider the regular hexagons $H_k$ that are centered at $x_{\tau (1)}$, and have radius *k* and one side parallel to the vector $x_{\tau (2)}-x_{\tau (1)}$, and proceed by induction on the radius $k\in \mathbb {N}$. To this aim, notice that the number of points of ${\mathcal {L}}_t$ contained in $H_k$ is $n_k:=1+3k+3k^2$. Assume that all the points $x_{\tau (i)}$, with $i\le n_k$, have been identified. We define $x_{\tau (1+n_k)}$ as the point $p\in {\mathcal {L}}_t\cap \ell _k$ such that $|p-x_{\tau (n_k)}|=1$ and $p\ne x_{\tau (n_k-1)}$, where $\ell _k$ denotes the line parallel to the vector $x_{\tau (2)}-x_{\tau (1)}$, and passing through the point $x_{\tau (n_k)}$. For $i\in (n_k+1, n_{k+1}]$, we then define $x_{\tau (i)}$ by clockwise numbering the points of ${\mathcal {L}}_t$ on the boundary of $H_k$ (see Fig. 1).

**Fig. 2:**
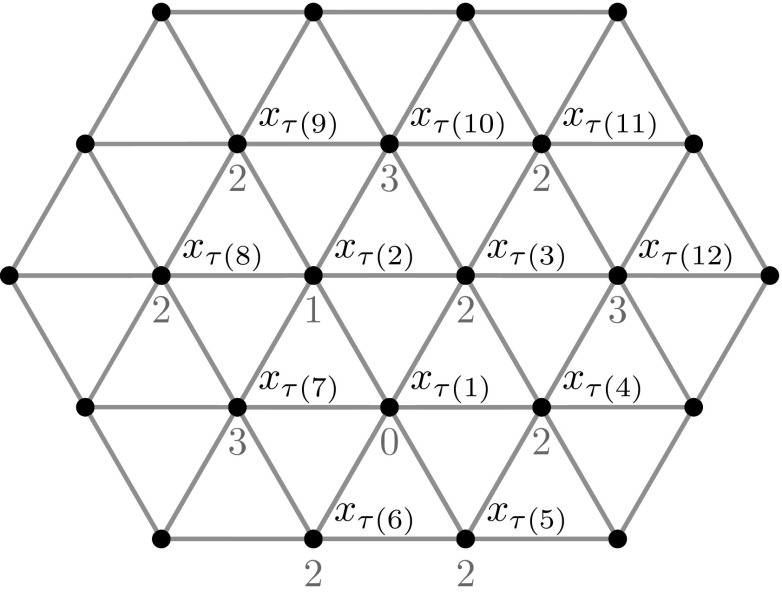
The first elements of ${\mathcal {L}}_t$ with respect to the order $\tau$ are shown with their weight assigned by the value of the function $\omega$ appearing below them

We will write $x<_{\mathcal {\tau }}y$ referring to the total order $\tau$ described above. A weight function $\omega$ is defined on ${\mathcal {L}}_t$ by the following

$$\begin{aligned} \omega (x):=|\{y\in {\mathcal {L}}_t \ : \ |x-y|=1 \text { and } y<_{\tau }x\}|, \end{aligned}$$

for every $x\in {\mathcal {L}}_t$. We observe that $\omega$ assumes value 0 at the point $x_{\tau (1)}$, value 1 at $x_{\tau (2)}$ (that is a point bonded to $x_{\tau (1)}$), and values 2 or 3 at all the other points in ${\mathcal {L}}_t$ (see Fig. 2). Furthermore, we have that

31 $$\begin{aligned} E(D_n)=-\sum _{i=1}^n\omega (x_{\tau (i)})\quad \text {for every }n\in \mathbb {N}. \end{aligned}$$

and that ${\mathcal {L}}_t=\{ x_{\tau (1)},x_{\tau (2)}\}\cup \Omega _2\cup \Omega _3$ with

$$\begin{aligned} \Omega _2:=\{x \in {\mathcal {L}}_t\,:\, \omega (x)=2\}\quad \text {and}\quad \Omega _3:=\{x \in {\mathcal {L}}_t \,:\,\omega (x)=3\}. \end{aligned}$$

Moreover, for every configuration $C_n$ we introduce a weight function $\omega _{C_n}$ defined by

32 $$\begin{aligned} \omega _{C_n}(x):=|\{y\in C_n \ : \ |x-y|=1 \text { and } y<_{\tau }x\}|, \end{aligned}$$

for every $x\in C_n$ (and thus depending on $C_n$). In this way $C_n$ can be rewritten as the union

$$\begin{aligned} C_n=\bigcup _{k=0}^3C_n^{k}\end{aligned}$$

where

33 $$\begin{aligned} C_n^{k}:=\{x \in C_n\,:\, \omega _{C_n}(x)=k\} \end{aligned}$$

for $k=0,\ldots ,3$. We notice that $\omega _{C_n}(x)\le \omega (x)$ for every $x\in C_n$ and that $|C_n^{0}|$ is the number of connected components of $C_n$.

In order to prove the isoperimetric inequality (13), we first express the energy, the perimeter, the edge perimeter, and the area of a regular configuration $C_n$ as a function of the cardinality of the sets $C_n^{k}$.

### Proposition 2.2

Let $C_n$ be a regular configuration in ${\mathcal {L}}_t$. Then

34 $$\begin{aligned}&E(C_n)=-|C_n^{1}|-2|C_n^{2}|-3|C_n^{3}|,\end{aligned}$$

35 $$\begin{aligned}&A(C_n)=|C_n^{2}|+2|C_n^{3}|,\end{aligned}$$

36 $$\begin{aligned}&P(C_n)=2|C_n^{1}|+|C_n^{2}|,\end{aligned}$$

37 $$\begin{aligned}&|\Theta (C_n)|=6|C_n^{0}|+4|C_n^{1}|+2|C_n^{2}|, \end{aligned}$$

for every $$n\in \mathbb {N}$$.

### Proof

Fix $n\in {\mathbb {N}}$, and let $C_n$ be a regular configuration in ${\mathcal {L}}_t$. In analogy to (31) there holds

$$\begin{aligned} E(C_n)=-\sum _{i=1}^n\omega _{C_n}(x_{i}). \end{aligned}$$

For $i=0,\ldots ,n-1$, denote by $C_{i}$ the subset of $C_n$ containing the first *i* points of $C_n$, according to the total order $\tau$. If $x_{\tau (i)}\in C_n^{0}$, then

38 $$\begin{aligned} A(C_i)-A(C_{i-1})=0,\quad P(C_i)-P(C_{i-1})=0\quad \text {and}\quad |\Theta (C_i)|-|\Theta (C_{i-1})|=6;\nonumber \\ \end{aligned}$$

if $$x_{\tau (i)}\in C_n^{1}$$, then

39 $$\begin{aligned} A(C_i)-A(C_{i-1})=0,\quad P(C_i)-P(C_{i-1})=2\quad \text {and}\quad |\Theta (C_i)|-|\Theta (C_{i-1})|=4;\nonumber \\ \end{aligned}$$

if $$x_{\tau (i)}\in C_n^{2}$$, then

40 $$\begin{aligned} A(C_i)-A(C_{i-1})=1,\quad P(C_i)-P(C_{i-1})=1\quad \text {and}\quad |\Theta (C_i)|-|\Theta (C_{i-1})|=2;\nonumber \\ \end{aligned}$$

whereas, if $x_{\tau (i)}\in C_n^{3}$, we have

41 $$\begin{aligned} A(C_i)-A(C_{i-1})=2,\quad P(C_i)-P(C_{i-1})=0\quad \text {and}\quad |\Theta (C_i)|-|\Theta (C_{i-1})|=0.\nonumber \\ \end{aligned}$$

In view of (38)–(41), we obtain (34)–(37). $\square$

We notice that from (34),(35), and (36) we also recover (28), which in turn, together with (37), yields

42 $$\begin{aligned} E(C_n)=-\frac{3}{2}A(C_n)-\frac{1}{4}|\Theta (C_n)| + \frac{3}{2}|C_n^{0}| \end{aligned}$$

for every configuration $C_n$. Moreover, from the equality

$$\begin{aligned} \sum _{i=0}^3 |C_n^{i}|=n, \end{aligned}$$

(35), and (36) it follows that

43 $$\begin{aligned} A(C_n)=2n -2|C^{0}_n| -P(C_n). \end{aligned}$$

Note that in particular if $C_n=D_n$ then $\omega _{C_n}(x)=\omega (x)$. Furthermore, $D_n^{0}=\{x_{\tau (1)}\}$, $D_n^{1}=\{x_{\tau (2)}\}$, $D_n^{2}=\Omega _2\cap D_n$, and $D_n^{3}=\Omega _3\cap D_n$. Therefore, (34)–(42) yield

44 $$\begin{aligned} E(D_n)&=-1-2|\Omega _2\cap D_n|-3|\Omega _3\cap D_n|, \end{aligned}$$

45 $$\begin{aligned} A(D_n)&=|\Omega _2\cap D_n|+2|\Omega _3\cap D_n|, \end{aligned}$$

46 $$\begin{aligned} P(D_n)&=2+|\Omega _2\cap D_n|, \end{aligned}$$

47 $$\begin{aligned} |\Theta (D_n)|&=10+2|\Omega _2\cap D_n|, \end{aligned}$$

and by (42) and (43) we obtain

$$\begin{aligned} E(D_n)=-\frac{3}{2}A(D_n)-\frac{1}{4}|\Theta (D_n)| + \frac{3}{2}, \end{aligned}$$

and

$$\begin{aligned} A(D_n)=2n -2-P(D_n) \end{aligned}$$

for every $$n>1$$.

### Proposition 2.3

The following assertions are equivalent and hold true for every connected configuration $C_n$:

(i) $$|\Theta (D_n)|\le |\Theta (C_n)|$$;
(ii) $$P(D_n)\le P(C_n)$$;
(ii) $$A(D_n)\ge A(C_n)$$.

### Proof

The first assertion follows directly from (30) and is equivalent to the second by (36) and (37). The second assertion is equivalent to the third by (29) and (30). $\square$

### Proof of Theorem 1.1

In this subsection, we prove Theorem 1.1 by characterizing the minimizers of EIP as the solutions of a discrete isoperimetric problem. We proceed in two steps.

*Step 1* We claim that

48 $$\begin{aligned} \sqrt{A(D_n)} = k_n P(D_n). \end{aligned}$$

Indeed, by (11), (12), (30), (44), there holds

49 $$\begin{aligned} \frac{\theta _n}{2}-3n=e_n=E(D_n)=-1-2|\Omega _2\cap D_n|-3|\Omega _3\cap D_n|. \end{aligned}$$

Equalities (12) and (47) yield

50 $$\begin{aligned} \theta _n= |\Theta (D_n)|= 10 +2|\Omega _2\cap D_n|. \end{aligned}$$

Theorefore, by (49) and (50), we have

51 $$\begin{aligned} |\Omega _2\cap D_n|= \frac{\theta _n}{2} -5, \end{aligned}$$

and

52 $$\begin{aligned} |\Omega _3\cap D_n|=-\frac{\theta _n}{2}+n +3. \end{aligned}$$

Claim (48) follows now by (45), (46), (51) and (52), and by observing that

$$\begin{aligned} \sqrt{A(D_n)}&= \displaystyle \sqrt{ |\Omega _2\cap D_n|+2|\Omega _3\cap D_n|} = \displaystyle \sqrt{ \theta _n/2 -5 +2(-\theta _n/2+n+3)}\\&=\displaystyle \sqrt{-\theta _n/2 + 2n +1 }=k_n(\theta _n/2 -3)=k_n(|\Omega _2\cap D_n|+2)=k_n P(D_n). \end{aligned}$$

Inequality (13) is a direct consequence of (48) and Proposition 2.3. By Proposition 2.3 we also deduce that the maximal area and the minimal perimeter among connected configurations are realized by $A(D_n)=-\theta _n/2 + 2n +1$ and $P(D_n)=\theta _n/2-3$, respectively.

*Step 2* We prove the characterization statement of Theorem 1.1. Let $C_n$ be a connected configuration satisfying

53 $$\begin{aligned} \sqrt{A(C_n)} = k_n P(C_n). \end{aligned}$$

We claim that $C_n$ is a minimizer. In fact, the claim follows from

$$\begin{aligned} |\Theta (D_n)|&\le |\Theta (C_n)|= 6n - 3A(C_n)\,-\, P(C_n)\,\\&=\, 6n\,-\,3(k_n)^2 (P(C_n))^2 \,-\,P(C_n)\\&\le 6n -3(k_n)^2 (P(D_n))^2 \,-\,P(D_n)\\&=\,6n\,-\,3A(D_n)- P(D_n)=|\Theta (D_n)| \end{aligned}$$

where we used (30) in the first inequality, (29) in the first and last equality, (28) in the second, (53) in the third, Proposition 2.3 in the second inequality, and (48) in the third equality.

Viceversa, let $M_n$ be a connected minimizer. By (10), (36), and (37), $P(M_n)=P(D_n)$; by (28), $A(M_n)=A(D_n)$. Thus (13) holds with the equality by (48). This concludes the proof of the theorem.

## Maximal Hexagons Associated to EIP Minimizers

In this section, we introduce the notion of maximal hexagons $H_{r_{M_n}}$ associated to minimizers $M_n$ and we provide a uniform lower estimate of $r_{M_n}$ in terms of *n* [see (69)].

Fix a minimizer $M_n$. Let ${\mathcal {H}}^{M_n}_s$ be the family of the configurations contained in $M_n$ that can be seen as translations in ${\mathcal {L}}_t$ of daisy configurations $D_{1 +3s + 3s^2}$ for some $s\in \mathbb {N}\cup \{0\}$, i.e.,

54 $$\begin{aligned} {\mathcal {H}}^{M_n}_s:=\{H_s\subset {\mathcal {L}}_t \ : \ H_s:=D_{1 +3s + 3s^2}+q \text { for some } q\in {\mathcal {L}}_t \text { and } H_s\subset M_n\},\qquad \end{aligned}$$

and choose $H_{r_{M_n}}$ to be a configuration in ${\mathcal {H}}^{M_n}_{r_{M_n}}$ where

55 $$\begin{aligned} r_{M_n}:=\max \{s\in \mathbb {N}\cup \{0\} \ : \ {\mathcal {H}}^{M_n}_s\ne \emptyset \}. \end{aligned}$$

We will refer to $H_{r_{M_n}}$ as the *maximal hexagon associated to* $M_n$. Notice that the number of atoms of $M_n$ contained in $H_{r_{M_n}}$ is

56 $$\begin{aligned} n(r_{M_n}):=1+3\,r_{M_n} +3\left( r_{M_n}\right) ^2. \end{aligned}$$

**Fig. 3:**
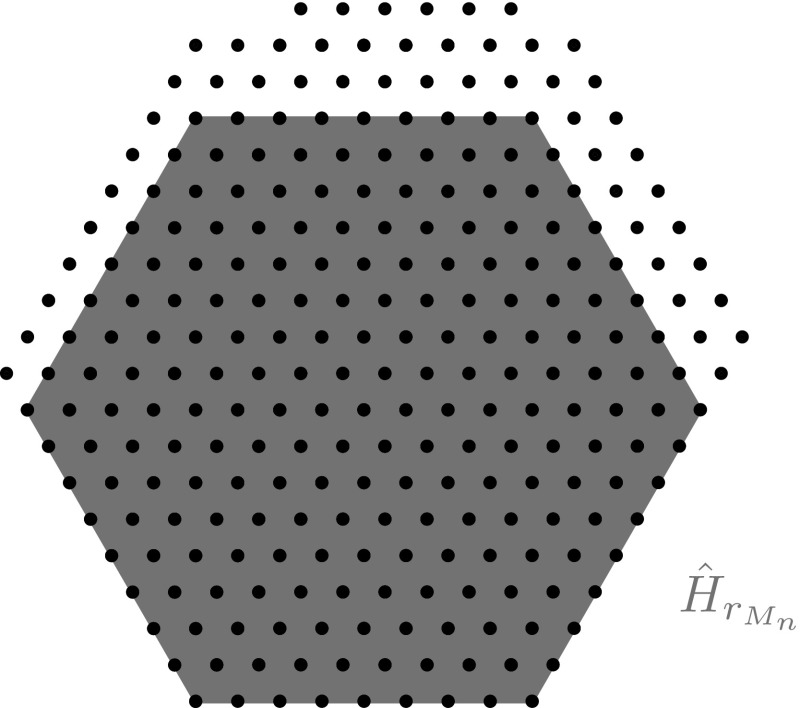
A minimizer $M_n$ is represented by the set of *dots* and its maximal hexagon $H_{r_{M_n}}$ is given by the intersection of $M_n$ with the regular hexagon ${\hat{H}}_{r_{M_n}}$ which is drawn in dark color (*blue*) (Color figure online)

In the following, we will often denote the minimal regular hexagon containing $H_{r_{M_n}}$ by ${\hat{H}}_{r_{M_n}}$ (see Fig. 3), i.e.,

$$\begin{aligned} {\hat{H}}_{r_{M_n}}:=F(H_{r_{M_n}}) \end{aligned}$$

Following the notation introduced in Sect. 2 in (33), we decompose $M_n$ as

$$\begin{aligned} M_n=\bigcup _{k=0}^3M_n^{k}. \end{aligned}$$

In the following proposition, we observe that if $n>6$, then there exists a non-degenerate maximal hexagon for every minimizer.

### Proposition 3.1

For $n\le 6$, then the maximal hexagon $H_{r_{M_n}}$ is degenerate for every minimizer $M_n$ of (2). If $n>6$, then the maximal radius $r_{M_n}$ of every minimizer $M_n$ of (2) satisfies $r_{M_n}\ge 1$.

### Proof

It is immediate to check that for $n=1$, $|M_n^{1}|=0$, and for $n=2$ or $n=3$, $|M_n^{1}|=1$. A direct analysis of the cases in which $n=4,5,6,$ shows that $2\ge |M_n^{1}|\ge 1$. It is also straightforward to observe that for $n=0,\ldots ,6$, there holds $r=0$.

We claim that for $n\ge 7$ the radius $r_{M_n}$ satisfies $r_{M_n}\ge 1$. Indeed, assume that $M_n$ is such that $r_{M_n}=0$. Then $M_n$ does not contain any hexagon with radius 1 and hence, for every $x\in M_n$ we have that

57 $$\begin{aligned} b(x)\le 5. \end{aligned}$$

Property (57) is equivalent to claiming that every element of $M_n$ contributes to the overall perimeter of $M_n$, and the contribution of each element is at least 1. Therefore,

$$\begin{aligned} P(M_n)\ge n. \end{aligned}$$

By Theorem 1.1, it follows that

58 $$\begin{aligned} \frac{\theta _n}{2}-3\ge n, \end{aligned}$$

which in turn by (12) implies

$$\begin{aligned} \sqrt{12n-3}-2\ge \lceil \sqrt{12n-3}\rceil -3\ge n, \end{aligned}$$

that is

$$\begin{aligned} n^2-8n+7\le 0, \end{aligned}$$

which finally yields $1\le n\le 7$. To conclude, it is enough to notice that for $n=7$, $\theta _n/2-3=6$, thus contradicting (58). $\square$

In view of Proposition 3.1 for every minimizer $M_n$ with $n>6$, we can fix a vertex $V_0$ of its (non-degenerate) hexagon ${\hat{H}}_{r_{M_n}}$ and denote by $V_1,\ldots ,V_5$ the other vertices of ${\hat{H}}_{r_{M_n}}$ numbered counterclockwise starting from $V_0$. For $k=0,\ldots ,4$, let us also denote by $s_k$ the line passing through the side of ${\hat{H}}_{r_{M_n}}$ with endpoints $V_k$ and $V_{k+1}$, and let $s_5$ be the line passing through $V_5$ and $V_0$.

In the following we will need to consider the number of *levels* of atoms in ${\mathcal {L}}_t$ around $H_{r_{M_n}}$ containing at least one element of $M_n$. Denote by $\mathbf{e }_k$ the outer unit normal to the side $s_k$ of ${\hat{H}}_{r_{M_n}}$ and define

59 $$\begin{aligned} \lambda _k:=\max \{j\in \mathbb {N}\,:\, s_k^{j}\cap M_n\ne \emptyset \} \end{aligned}$$

where $s_k^{j}$ are the lines of the lattice ${\mathcal {L}}_t$ parallel to $s_k$ and not intersecting $H_{r_{M_n}}$, namely

$$\begin{aligned} s_k^{j}:=s_k +\frac{\sqrt{3}}{2}j\mathbf{e }_k \end{aligned}$$

for $j\in \mathbb {Z}$. Let also $\pi _k$ be the open half-plane with boundary $s_k$ and not intersecting the interior of ${\hat{H}}_{r_{M_n}}$.

We first show that $M_n$ satisfies a connectedness property with respect to the directions determined by the lattice ${{\mathcal {L}}}_t$. To this purpose, we introduce the notion of *3-convexity with respect to* ${\mathcal {L}}_t$.

### Definition 3.2

We recall that

$$\begin{aligned} {\varvec{t}}_1:=\left( 1,0\right) ,\quad {\varvec{t}}_2:=\left( \frac{1}{2},\frac{1}{2\sqrt{3}}\right) ,\quad \text {and define}\quad {\varvec{t}}_3:={\varvec{t}}_2-{\varvec{t}}_1. \end{aligned}$$

We say that a set $S\subset {{\mathcal {L}}}_t$ is *3-convex* if for every $p,q\in S$ such that $q:=m{\varvec{t}}_i\,+\,p$ for some $m\in \mathbb {N}$ and $i\in \{1,2,3\}$ one has that $q':=m'{\varvec{t}}_i\,+\,p\in S$ for every integer $m'\in (0,m)$. Furthermore, we refer to the lines

$$\begin{aligned} \ell ^p_i:=\{ q\in \mathbb {R}^2\,:\, q=r{\varvec{t}}_i\,+\,p \text { for some } r\in \mathbb {R}\} \end{aligned}$$

as the *lines of the lattice* ${\mathcal {L}}_t$ *at* *p*.

Note that by Definition 3.2 a set *S* is 3-convex if there is no line $\ell ^p_i$ of the lattice ${\mathcal {L}}_t$ at a point $p\in {\mathcal {L}}_t\setminus S$ that is separated by *p* in two half-lines both containing points of the set *S*.

### Proposition 3.3

Let $M_n$ be a minimizer. Then $M_n$ is 3-convex.

### Proof

For the sake of contradiction assume that the minimizer $M_n$ is a not 3-convex. Then there exist a point $p\in {\mathcal {L}}_t\setminus M_n$ and $i\in \{1,2,3\}$ such that the line $\ell ^p_i$ (see Definition 3.2) is divided by *p* in two half-lines both containing points of $M_n$. We claim that we can rearrange the *n* points of $M_n$ in a new 3-convex configuration ${\tilde{M}}_n$ such that $|\Theta ({\tilde{M}}_n)|<|\Theta (M_n)|$ thus contradicting optimality.

Denote for simplicity $\ell _0:=\ell ^p_i$ and let $\ell _1,\ldots ,\ell _m$ be all the other lines parallel to $\ell _0$ that intersect $M_n$. Furthermore, let $c_k=|M_n\cap \ell _k|$ for $k=1,\ldots ,m$. Starting from the elements of the sequence $\{c_k\}$, we rearrange them in a decreasing order, constructing another set $\{d_k\}$ with the property that $d_0\ge d_1\ge \cdots \ge d_m$. Finally, we separate the elements of $\{d_k\}$ having odd indexes from those having even indexes and we rearrange them in a new set $\{f_k\}$ obtained by first considering the elements of $\{d_k\}$ with even indexes, in decreasing order with respect to their indexes, and then the elements of $\{d_k\}$ having odd indexes, with increasing order with respect to their indexes. The set $\{f_k\}$ constructed as above has the property that the two central elements have the maximal value, and the values of the elements decrease in an alternated way by moving toward the sides of the ordered set. Let ${\bar{k}}$ be the index corresponding to the central element of the set $\{f_k\}$, if *m* is even, and to the maximum between the two central elements of $\{f_k\}$, if *m* is odd.

As an example, if we start with a set $\{c_k\}=\{9,4,2,5,3,1,17\}$, the sequence $\{d_k\}$ is given by $\{17,9,5,4,3,2,1\}$ and the sequence $\{f_k\}$ by $\{1,3,5,17,9,4,2\}$. Here ${\bar{k}}=4$.

Fix a point $P_{{\bar{k}}}\in {{\mathcal {L}}}_t$ and an angular sector *S* of amplitude $2\pi /3$, with vertex in $P_{{\bar{k}}}$, whose sides $\sigma _1$ and $\sigma _2$ lay on the two lines departing from $P_{{\bar{k}}}$ which are not parallel to $\ell _0$. Consider the points $P_0,\ldots ,P_{{\bar{k}}-1}\in \sigma _1\cap M_n$, such that

$$\begin{aligned} |P_k-P_{{\bar{k}}}|={\bar{k}}-k\quad \text {for }k=0,\ldots {\bar{k}}-1. \end{aligned}$$

Analogously, consider the points $P_{{\bar{k}}+1},\ldots ,P_m\in \sigma _2\cap M_n$, satisfying

$$\begin{aligned} |P_k-P_{{\bar{k}}}|=k-{\bar{k}}\quad \text {for }k={\bar{k}}+1,\ldots ,m. \end{aligned}$$

For $k=0,\ldots ,m$, let ${\tilde{\ell }}_k$ be the line parallel to $\ell _0$ and passing through $P_k$. To construct the set ${\tilde{M}}_n$, we consider $f_k$ consecutive points on each line ${\tilde{\ell }}_k$, starting from $P_k$. We note that $|{\tilde{M}}_n|=|M_n|=n$, the number of bonds in each line parallel to $\ell _0$ has increased. On the other hand, the number of bonds between different lines has not decreased. Indeed, given two parallel lines with *a* and *b* points, respectively, the maximal number of bonds between these two lines is either 2*a* if $a<b$, or $2a-1$ if $a=b$. This maximal value is achieved by construction by the modified configuration. Hence,

$$\begin{aligned} |\Theta ({\tilde{M}}_n)|< |\Theta (M_n)|, \end{aligned}$$

providing a contradiction to the optimality of $M_n$. $\square$

Since every minimizer $M_n$ is 3-convex, the quantity $\lambda _k$ introduced in (59) for $k=0,\ldots ,5$ provides the number of non-empty levels of atoms in $M_n\cap \pi _k$ for $n>6$. In fact, by the definition of $\tau$ each partially full level contains at least one point in $(M_n^{1}\cup M_n^{2})\setminus H_{r_{M_n}}$. Hence,

60 $$\begin{aligned} \sum _{k=0}^5\lambda _k\le |M_n^{1}\setminus H_{r_{M_n}}|+|M_n^{2}\setminus H_{r_{M_n}}|. \end{aligned}$$

On the other hand,

61 $$\begin{aligned} 2|M_n^{1}\setminus H_{r_{M_n}}|+|M_n^{2}\setminus H_{r_{M_n}}|=P(M_n)-P(H_{r_{M_n}})=p_n-6\,r_{M_n}. \end{aligned}$$

Therefore, by (60) and (61),

62 $$\begin{aligned} \sum _{k=0}^5\lambda _k\le p_n-6\,r_{M_n}. \end{aligned}$$

In the remaining part of this section, we provide a characterization of the geometry of $M_n\setminus H_{r_{M_n}}$ for $n>6$, by subdividing this set into *good* polygons $P_k$ and *bad* polygons $T_k$, and by showing that the cardinality of $M_n\setminus H_{r_{M_n}}$ is, roughly speaking, of the same order of magnitude as the one of the union of *good* polygons.

Given a minimizer $M_n$ and its maximal hexagon $H_{r_{M_n}}$, we denote by $H_{r_{M_n}+1}$ the hexagon with side $r_{M_n}+1$ and having the same center as $H_{r_{M_n}}$. In the following, we denote the hexagon containing $H_{r_{M_n}+1}$ by

$$\begin{aligned} {\hat{H}}_{r_{M_n}+1}:=F(H_{r_{M_n}+1}). \end{aligned}$$

We first show that, by the optimality of $H_{r_{M_n}}$, there exists an angular sector of $2\pi /3$, and centered in one of the vertices of ${\hat{H}}_{r_{M_n}+1}$, which does not intersect $M_n$. To this end, we denote by $V_i'$, $i=0,\ldots ,5$ the vertices of the hexagon ${\hat{H}}_{r_{M_n}+1}$, with the convention that $V_i'$ lies on the half-line starting from the center of $H_{r_{M_n}}$ and passing through $V_i$.

### Lemma 3.4

Let $M_n$ be a minimizer with $r_{M_n}>0$. Then

$${ (i)}$$
The hexagon ${\hat{H}}_{r_{M_n}+1}$ presents at least a vertex, say $V'_j$ with $j\in \{0,\ldots ,5\}$, that does not belong to $M_n$.
$${ (ii)}$$
There exists $k\in \{0,\ldots ,5\}$ such that the open angular sector $S_k$ of amplitude $2\pi /3$, centered in $V'_k$, and with sides $s^1_k$ and $s^1_{k-1}$ *(*with the convention that $s^1_{-1}:=s^1_{5}$ *)* is such that $\overline{S_k}\cap M_n=\emptyset$.
$${ (iii)}$$
Every translation ${\hat{H}}$ of ${\hat{H}}_{r_{M_n}+1}$ by a vector ${\mathbf{t }}:=n{\varvec{t}}_1+m{\varvec{t}}_2$ with $n,m\in \mathbb {Z}$ that has a vertex $v\not \in M_n$ admits a vertex $w\not \in M_n$ (possibly different from *v*) and an open angular sector *S* of amplitude $2\pi /3$ and centered in *w* such that ${\overline{S}}\cap M_n=\emptyset$.

### Proof

We begin by showing assertion (*i*). In view of the maximality of $H_{r_{M_n}}$ there exists a point $p\in {\mathcal {L}}_t$ on the boundary of ${\hat{H}}_{r_{M_n}+1}$ such that $p\notin M_n$. Either *p* is already a vertex of ${\hat{H}}_{r_{M_n}+1}$ or *p* is an internal point on the side of ${\hat{H}}_{r_{M_n}+1}$ parallel to $s_j$ for some *j*. In this latter case, by the 3-convexity of $M_n$, either $V_{j}'$ or $V_{j+1}'$ does not belong to $M_n$ and hence, also in this case assertion (*i*) holds true.

We now denote by $V_{j}'$ the missing vertex of the hexagon ${\hat{H}}_{r_{M_n}+1}$ and prove assertion (*ii*). Let us consider the two half-lines in which $V_j'$ divides the line $s_j^1$. By the 3-convexity of $M_n$, at least one of them does not intersect $M_n$. Analogously, if we consider the two half-lines in which $V'_j$ divides the line $s^1_{j-1}$, by the 3-convexity of $M_n$ at least one of them does not intersect $M_n$. Finally, if we consider the line $s'$ passing through the center of $H_{r_{M_n}}$ and $V_j'$, the 3-convexity of $M_n$ implies that the points of $s'$ whose distance from the center of $H_{r_{M_n}}$ is bigger than $r_{M_n}+1$ do not belong to $M_n$. In view of the geometric position of such three half-lines departing from $V_j'$, we can conclude that the claim holds true by using once again the 3-convexity of $M_n$.

Let us conclude by observing that assertion (*iii*) follows by a similar argument to the one employed to prove assertion (*ii*). If the center of ${\hat{H}}$ is in $M_n$, then the same argument works and we can chose $w=v$. If the center of ${\hat{H}}$ is not in $M_n$, then the line passing through the missing vertex *v* and the center of ${\hat{H}}$ does not intersect $M_n$ outside ${\hat{H}}$ either for *v* or for the opposite vertex *w* with respect to the center of ${\hat{H}}$. $\square$

In the following, we assume without loss of generality that the vertex $V_0$ has been chosen so that the index *k* in assertion (*ii*) of Lemma 3.4 is 0. Therefore, by assertion (*ii*) of Lemma 3.4 we obtain that the open angular sector $S_0$ of $2\pi /3$, centered in $V'_0$, and with sides $s^1_0$ and $s^1_{5}$ is such that $\overline{S_0}\cap M_n=\emptyset$.

**Fig. 4:**
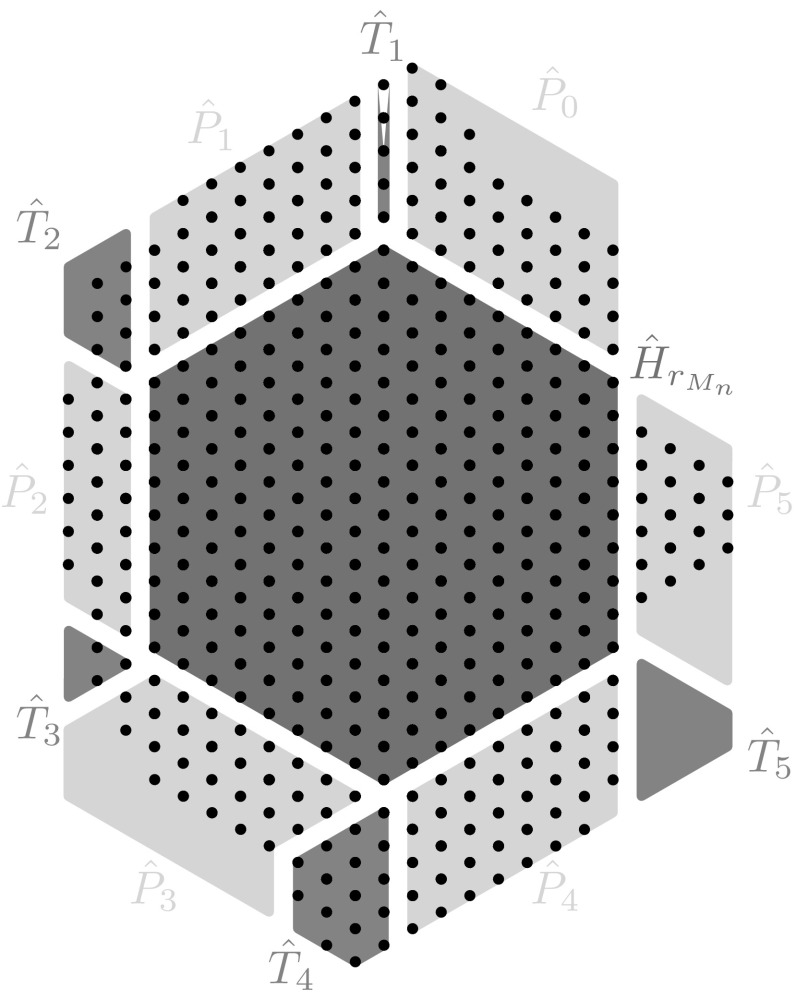
Representation of the region ${\hat{R}}$ given by the union of the polygons ${\hat{P}}_j$ with $j=0,\ldots ,5$ drawn in the lightest color (*yellow*) and the polygons ${\hat{T}}_j$ with $j=1,\ldots ,5$ drawn in the middle color (*green*). Note that this picture has a mere illustrative purpose (the configuration is not a EIP minimizer) (Color figure online)

Let us use the definition of the levels $\lambda _k$ for $k=0,\ldots ,5$ introduced in (59) to define a region ${\hat{R}}$ that contains all extra points of $M_n$, i.e., points of $M_n$ not contained in $H_{r_{M_n}}$. We already know that we can take ${\hat{R}}\subset (\mathbb {R}^2\setminus {\hat{H}}_{r_{M_n}})\cap (\mathbb {R}^2\setminus S_0)$. We define the region ${\hat{R}}$ as follows (see Fig. 4):

63 $$\begin{aligned} {\hat{R}}:=\Big (\bigcup _{j=0}^5 {\hat{P}}_j\Big )\bigcup \Big (\bigcup _{j=1}^5 {\hat{T}}_j\Big ) \end{aligned}$$

The set ${\hat{P}}_0$ in (63) is the polygon delimited by the lines $s_5,s_0^{1},s_0^{\lambda _0},s_5^{-r+1}$ and the sets ${\hat{P}}_k$ in (63) is defined by

$$\begin{aligned} {\hat{P}}_k:={\left\{ \begin{array}{ll} {\hat{P}}_k^{1}(\lambda _k)&{}\text {if }\lambda _k\le \lambda _{k-1}+1,\\ {\hat{P}}_k^{1}(\lambda _k-\lambda _{k-1}+1)\cup {\hat{P}}_k^{2}(\lambda _k-\lambda _{k-1}+1)&{}\text {if }\lambda _k>\lambda _{k-1}+1, \end{array}\right. } \end{aligned}$$

for every $k=1,\ldots ,5$, where for every $a\in [-2\,r_{M_n},2\,r_{M_n}]$ we denote by ${\hat{P}}_k^{1}(a)$ the polygon contained between $s_k^{1}$, $s_k^{a}$, $s_{k+1}$, $s_{k+1}^{-r+1}$, and by ${\hat{P}}_k^{2}(a)$ the set delimited by $s_k^{a}$, $s_k^{\lambda _k}$, $s_{k-1}^{\lambda _{k-1}-r+1}$, $s_{k-1}^{\lambda _{k-1}}$. Finally the sets ${\hat{T}}_k$ are the *region* between ${\hat{P}}_{k-1}$ and ${\hat{P}}_k$ or, more precisely,

64 $$\begin{aligned} {\hat{T}}_k:=&\{x\in R:\,x\in s_{k-1}^{j_{k-1}}\cap s_{k}^{j_{k}},\,\text {with }1\le j_{k-1}\le \lambda _{k-1},\,1\le j_k\le \lambda _k,\,j_{k-1}\ge j_k\,\nonumber \\&\quad \text {and, if }\lambda _{k-1}>\lambda _{k-2}+1,\, j_{k-1}\le j_k+\lambda _{k-1}-\lambda _{k-2}\}. \end{aligned}$$

Note that ${\hat{T}}_1$ by definition (64) reduces to a segment contained in the line $s_2^{-r_{M_n}}$ such that

65 $$\begin{aligned} |T_1|=\min \{\lambda _0,\lambda _1\}. \end{aligned}$$

Furthermore, we consider the configurations $P_k:=\hat{P_k}\cap {\mathcal {L}}_t$ for $k=0,\ldots ,5$, $T_k:=\hat{T_k}\cap {\mathcal {L}}_t$ for $k=1,\ldots ,5$, and $R:={\hat{R}}\cap {\mathcal {L}}_t$. We notice that $M_n\subset H_{r_{M_n}}\cup R$ and that

$$\begin{aligned} n=|H_{r_{M_n}}|+|R|-|R\setminus M_n|, \end{aligned}$$

where $$|H_{r_{M_n}}|=1+3\,r_{M_n}+3\left( r_{M_n}\right) ^2$$, and

$$\begin{aligned} |R|=\sum _{k=0}^5|P_k|+\sum _{k=1}^5 |T_k|= r_{M_n}\sum _{k=0}^{5}\lambda _k+\sum _{k=1}^5 |T_k| \end{aligned}$$

where in the last equality we used that $|P_k|=r_{M_n}\lambda _k$ for $k=0,\ldots ,5$. Furthermore, for every $x\in R$ and every $k=0,\ldots ,5$ there exists $j_k\in [-\lambda _{k'}-2r,\lambda _k]$ with $k':=(k+3)_{\mathrm{mod }\, 6}$ and $k'\in \{0,\ldots ,5\}$ such that $x\in s_k^{j_k}$. Hence, in particular, every $x\in R$ is uniquely determined by a pair of indexes $(j_k,j_{k'})$, with $k'\ne k+3$ in $\mathbb {Z}_6$.

### Proposition 3.5

Let ${\mathcal {H}}$ be the family of the configurations that can be seen as translations in ${\mathcal {L}}_t$ of the daisy configuration $D_{1 +3s + 3s^2}$ for $s:=r_{M_n}+1$ and that are contained in $H_{r_{M_n}}\cup R$, i.e.,

$$\begin{aligned} {\mathcal {H}}:=\{H\subset H_{r_{M_n}}\cup R \,:\, H=D_{1 +3s + 3s^2}+q \text { for } s:=r_{M_n}+1 \text { and some } q\in {\mathcal {L}}_t\}. \end{aligned}$$

Then there holds

$$\begin{aligned} |R\setminus M_n|\ge |{\mathcal {H}}|. \end{aligned}$$

### Proof

Let $h:=|{\mathcal {H}}|$. We show by induction on $m=1,\ldots ,h$ that for every family ${\mathcal {H}}_m\subset {\mathcal {H}}$ with $|{\mathcal {H}}_m|=m$, there exists a set $V_{{\mathcal {H}}_m}\subset R\setminus M_n$ with $|V_{{\mathcal {H}}_m}|=m$, such that the correspondence that associates to each $v\in V_{{\mathcal {H}}_m}$ a hexagon $H\in {\mathcal {H}}_m$ if *v* is a vertex of ${\hat{H}}:=F(H)$, is a bijection.

We remark that the thesis will follow once we prove the assertion for $m=h$. The claim holds for $m=1$ by reasoning in the same way as in the first assertion of Lemma 3.4. Assume now that the claim is satisfied for $m={\bar{m}}$. Consider a family ${\mathcal {H}}_{{\bar{m}}+1}=\{H_1,\ldots ,H_{{\bar{m}}+1}\}\subset {\mathcal {H}}$, and the polygon

$$\begin{aligned} {\mathcal {P}}_{{\bar{m}}+1}:=\displaystyle \bigcup _{i=1}^{{\bar{m}}+1}H_i\subset H_{r_{M_n}}\cup R. \end{aligned}$$

Furthermore, let us define

$$\begin{aligned} \hat{{\mathcal {P}}}_{{\bar{m}}+1}:=F({\mathcal {P}}_{{\bar{m}}+1}). \end{aligned}$$

We subdivide the remaining part of the proof into 4 steps.

*Step 1* There exists a vertex ${\tilde{v}}$ of $\hat{{\mathcal {P}}}_{{\bar{m}}+1}$ that is not in $M_n$. Indeed, if all vertices of $\hat{{\mathcal {P}}}_{{\bar{m}}+1}$ belong to $M_n$, by 3-convexity${\mathcal {P}}_{{\bar{m}}+1}\subset M_n$, and hence $H_{{\bar{m}}+1}\subset {\mathcal {P}}_{{\bar{m}}+1}\subset M_n$, which would contradict the maximality of $r_{M_n}$.

*Step 2* By assertion (*iii*) of Lemma 3.4 there exists a vertex *w* of $\hat{{\mathcal {P}}}_{{\bar{m}}+1}$ not in $M_n$ and an open angular sector *S* centered in *w*, amplitude $2\pi /3$, and sides $\sigma _1,\sigma _2\subset {\mathcal {L}}_t$ such that ${\bar{S}}\cap M_n=\emptyset$.

*Step 3* There exists a vertex *v* of $\hat{{\mathcal {P}}}_{{\bar{m}}+1}$ that is not in $M_n$ and that corresponds to an interior angle of $\hat{{\mathcal {P}}}_{{\bar{m}}+1}$ of $2\pi /3$. In fact, $\hat{{\mathcal {P}}}_{{\bar{m}}+1}$ can have vertices with angles of $2\pi /3$, $4\pi /3$, and $5\pi /3$ only. If the vertex *w* detected in Step 2 corresponds to an angle of $2\pi /3$, there is nothing to prove. If *w* corresponds to an angle of $4\pi /3$ or $5\pi /3$, then we have two cases.

**Case 1** The intersection between *S* and the closure of $\hat{{\mathcal {P}}}_{{\bar{m}}+1}$ is empty. Then, for every $j=1,2$, there exists $v_j\in \sigma _j$ such that the segment with endpoints *w* and $v_j$ denoted by $wv_j$ is contained in $\partial \hat{{\mathcal {P}}}_{{\bar{m}}+1}$ and $v_j$ is a vertex of $\hat{{\mathcal {P}}}_{{\bar{m}}+1}$. Furthermore, $v_j\notin M_n$ because $v_j\in S$, and $v_j$ is associated to an angle of $2\pi /3$, since $S\cap \bar{{\mathcal {P}}}_{{\bar{m}}+1}=\emptyset$. The proof follows by taking $v=v_1$.

**Case 2** The intersection between *S* and the closure of $\hat{{\mathcal {P}}}_{{\bar{m}}+1}$ is nonempty. Without loss of generality, we can assume that the two sides of the angular sector *S* are given by

$$\begin{aligned} \sigma _1=\Big \{(\alpha ,\beta )\in {\mathbb {R}}^2:\,\beta =\alpha {\varvec{t}}_1+ w,\,\alpha >0 \Big \} \end{aligned}$$

and

$$\begin{aligned} \sigma _2=\Big \{(\alpha ,\beta )\in {\mathbb {R}}^2:\,\beta =-\alpha {\varvec{t}}_2+ w,\,\alpha >0\Big \}. \end{aligned}$$

Define

$$\begin{aligned} \sigma _1^k:=\sigma _1-\frac{\sqrt{3}}{2}k\,(0,1)\quad \text {and}\quad \sigma _2^k:=\sigma _2+k\,{\varvec{t}}_1, \end{aligned}$$

for $k\in \mathbb {N}$. Since ${\mathcal {P}}_{{\bar{m}}+1}\cap S$ is bounded, we can find

$$\begin{aligned} k_1:=\max \{k\in \mathbb {N}:\,\sigma _1^k\cap {\mathcal {P}}_{{\bar{m}}+1}\cap S\ne \emptyset \} \end{aligned}$$

and

$$\begin{aligned} k_2:=\max \{k\in \mathbb {N}:\,\sigma _2^k\cap {\mathcal {P}}_{{\bar{m}}+1}\cap S\ne \emptyset \}. \end{aligned}$$

For $j=1,2$, the intersection $\sigma _j^{k_j}\cap \partial \hat{{\mathcal {P}}}_{{\bar{m}}+1}\cap S$ is a segment with at least one endpoint $v\in S$ corresponding to a vertex of $\partial \hat{{\mathcal {P}}}_{{\bar{m}}+1}$ associated to an angle of $2\pi /3$.

*Step 4* Let *v* be the vertex provided by Step 3. Then, there exists a unique ${\hat{H}}_{{\bar{j}}}\in {\mathcal {H}}_{{\bar{m}}+1}$ having *v* among its vertices. By the induction hypothesis on $\{{\hat{H}}_1,\ldots ,{\hat{H}}_{{\bar{m}}+1}\}\setminus \{{\hat{H}}_{{\bar{j}}}\}$ there exists a family of vertices $\{v_j\}_{j=1,\ldots ,{\bar{m}}+1,\,j\ne {\bar{j}}}\subset R\setminus M_n$ such that $v_j$ is a vertex of ${\hat{H}}_j$ and for every $i\ne j$, $v_j$ is not a vertex of ${\hat{H}}_i$. The thesis follows then by setting $v_{{\bar{j}}}=v$, and by taking $V_{{\mathcal {H}}_{{\bar{m}}+1}}=\{v_1,\ldots ,v_{{\bar{m}}+1}\}$. $\square$

**Fig. 5:**
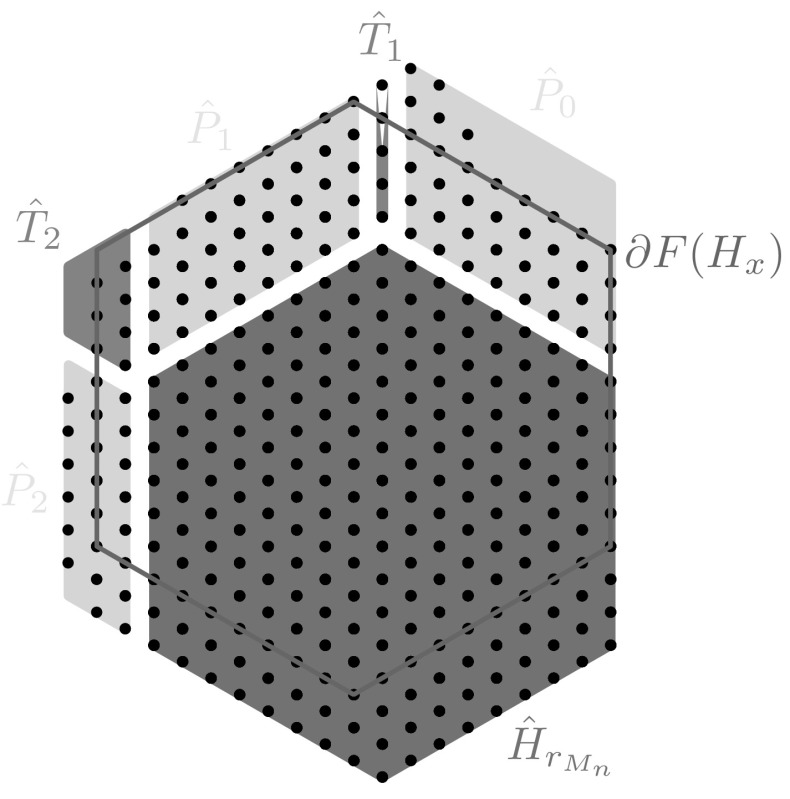
The region ${\hat{U}}_2$ is shown and the boundary $\partial F(H_x)$ of a hexagon $H_x\in {\mathcal {H}}_2$ with vertex $x\in T_2$ is represented by a continuous (*red*) line. Note that this picture has a mere illustrative purpose (the configuration is not a EIP minimizer) (Color figure online)

In view of Proposition 3.5 in order to estimate from below the cardinality of $R\setminus M_n$, it suffices to estimate the cardinality of ${\mathcal {H}}$. To this end, we denote in the following by ${\hat{U}}_k$ the closure of the region in $\mathbb {R}^2$ containing $H_{r_{M_n}}$ and delimited, respectively, by $s_{3}$, $s_{4}$, and $s_{5}$ for $k=2$, $s_{4}$, $s_{5}$, and $s_{0}$ for $k=3$, $s_{5}$, $s_{0}$, and $s_{1}$ for $k=4$, and $s_{0}$, $s_{1}$, and $s_{2}$ for $k=5$. Notice that $T_k\subset {\hat{U}}_k$ (see Fig. 5).

### Lemma 3.6

There holds

66 $$\begin{aligned} |{\mathcal {H}}|\ge \sum _{j=2}^5 |T_j|-\lambda _1-2\lambda _2-2\lambda _3-2\lambda _4-\lambda _5+4. \end{aligned}$$

### Proof

For notational simplicity we will omit in the rest of this proof the dependence of the radius $r_{M_n}$ on the minimizer $M_n$. We begin by noticing that

67 $$\begin{aligned} |{\mathcal {H}}|\ge \sum _{k=2}^5|{\mathcal {H}}_k| \end{aligned}$$

where

$$\begin{aligned} {\mathcal {H}}_k:=\{H\in {\mathcal {H}}:\, H\subset {\hat{U}}_k \text { and has a vertex in } T_k\} \end{aligned}$$

for $k=2,3,4,5$. We claim that

68 $$\begin{aligned} |{\mathcal {H}}_k|\ge |T_k|-\lambda _k-\lambda _{k-1}+1 \end{aligned}$$

and we observe that (66) directly follows from (67) and (68).

The rest of the proof is devoted to show (68). Let $x\in T_k$ and consider $(j_k,j_{k-1},j_{k-2})$ such that $x\in s_{k}^{j_k}\cap s_{k-1}^{j_{k-1}}\cap s_{k-2}^{j_{k-2}}$. In the following, we identify *x* with the triple of indexes $(j_k,j_{k-1},j_{k-2})$, and we write $x=(j_k,j_{k-1},j_{k-2})$. Let $H_x$ be the hexagon with vertices *x*,

$$\begin{aligned}&v_1:=(j_k-(r+1),j_{k-1},j_{k-2}+(r+1)),\\&v_2:=(j_k-2(r+1),j_{k-1}-(r+1),j_{k-2}+(r+1)),\\&v_3:=(j_k-2(r+1),j_{k-1}-2(r+1),j_{k-2}),\\&v_4:=(j_k-(r+1),j_{k-1}-2(r+1),j_{k-2}-(r+1)),\\&v_5:=(j_k,j_{k-1}-(r+1),j_{k-2}-(r+1)) \end{aligned}$$

(see Fig. 5 for an example of an hexagon $H_x\in {\mathcal {H}}_2$ with $x\in T_2$).

$H_x$ is contained in ${\hat{U}}_k$ if for every $j=0,\ldots ,5$ there holds $v_j\in {\hat{U}}_k$. This latter condition is equivalent to checking that the following inequalities are satisfied

$$\begin{aligned}&j_k-2(r+1)\ge -2r,\quad j_k\le \lambda _k,\\&j_{k-1}-2(r+1)\ge -2r,\quad j_{k-1}\le \lambda _{k-1},\\&j_{k-2}-(r+1)\ge -2r,\quad j_{k-2}+(r+1)\le \lambda _{k-2}. \end{aligned}$$

Hence, if $x=(j_j,j_{k-1},j_{k-2})\in T_k$ is such that

$$\begin{aligned}&2\le j_k\le \lambda _k,\\&2\le j_{k-1}\le \lambda _{k-1},\\&-r+1\le j_{k-2}\le \lambda _{k-2}-(r+1), \end{aligned}$$

then $H_x\subset {\hat{U}}_k$. By the definition of the sets $T_k$ [see (64)], the previous properties are fulfilled by every $x\in T_k$, apart from those points belonging to the portion of the boundary of ${\hat{T}}_k$ which is adjacent either to ${\hat{P}}_{k-1}$ or to ${\hat{P}}_k$. Denoting by ${\tilde{T}}_k$ this latter set, claim (68) follows once we observe that

$$\begin{aligned} |{\tilde{T}}_k|=|T_k|-\lambda _k-\lambda _{k-1} + 1. \end{aligned}$$

$$\square $$

Moving from Proposition 3.5 and Lemma 3.6, we deduce the lower estimate on the maximal radii $r_{M_n}$ of the minimizers $M_n$ of (2).

### Proposition 3.7

Let $M_n$ be a minimizer of (2) with maximal radius $r_{M_n}$. Then

69 $$\begin{aligned} r_{M_n}\ge \frac{{\left\lceil {\alpha _n}\right\rceil }}{6}-2-\frac{1}{6}\sqrt{{\left\lceil {\alpha _n}\right\rceil }^2-(\alpha _n)^2+75} \end{aligned}$$

with

70 $$\begin{aligned} \alpha _n:=\sqrt{12n-3}\,. \end{aligned}$$

### Proof

For the sake of notational simplicity, we will omit in the rest of this proof the dependence of the maximal radius $r_{M_n}$ from $M_n$. By Proposition 3.5 and Lemma 3.6 we have

$$\begin{aligned} |R\setminus M_n|\ge \sum _{j=2}^5 |T_j|-\lambda _1-2\lambda _2-2\lambda _3-2\lambda _4-\lambda _5+4, \end{aligned}$$

and so, by (62) and (65), we obtain

$$\begin{aligned}&n=|H_{r_{M_n}}|+|R|-|R\setminus M_n|\\&\quad \le 1+3r^2+3r+\sum _{j=0}^5|P_j|+\sum _{j=1}^5 |T_j|-\sum _{j=2}^5|T_j|+\lambda _1+2\lambda _2+2\lambda _3+2\lambda _4+\lambda _5-4\\&\quad \le 1+3r^2+3r+r\sum _{j=0}^5 \lambda _j+|T_1|+\lambda _1+2\lambda _2+2\lambda _3+2\lambda _4+\lambda _5-4\\&\quad \le 1+3r^2+3r+(r+2)\sum _{j=0}^5 \lambda _j-4\\&\quad \le 1+3r^2+3r+(r+2)(p_n-6r)-4=-3r^2+(p_n-9)r+2p_n. \end{aligned}$$

Thus, the maximal radius satisfies the following inequality:

71 $$\begin{aligned} 3r^2-(p_n-9)r+n-2p_n\le 0. \end{aligned}$$

Estimate (69) follows from (71) by solving (71) with respect to *r* and recalling that $p_n=\theta _n/2-3$ by Theorem 1.1 and $\theta _n=2{\left\lceil {\alpha _n}\right\rceil }$ by (12). $\square$

A direct consequence of (69) is the upper bound on the Hausdorff distance between the sets $M_n$ and $H_{r_{M_n}}$ introduced in Corollary 1.3.

### Proof of Corollary 1.3

Let $M_n$ be a minimizer. We assume with no loss of generality that $n>6$ so that by Proposition 3.1 the maximal hexagon $H_{r_{M_n}}$ is not degenerate. Then

$$\begin{aligned} d_{{\mathcal {H}}}(M_n, H_{r_{M_n}})\le \max _{i=0,\ldots ,5}\lambda _i. \end{aligned}$$

Therefore, by (62) and (70) we obtain that

$$\begin{aligned} {d_{{\mathcal {H}}}(M_n, H_{r_{M_n}})}&\le p_n-6\,r_{M_n}\\&\le 9+\sqrt{{\left\lceil {\alpha _n}\right\rceil }^2-(\alpha _n)^2+75}\\&=\sqrt{{\left\lceil {\alpha _n}\right\rceil }^2-(\alpha _n)^2}+\mathrm{O}(1)\\&\le \sqrt{2{\left\lceil {\alpha _n}\right\rceil }}+\mathrm{O}(1)\le \sqrt{2}\sqrt{\sqrt{12n-3}+1}+\mathrm{O}(1)\\&\le 2\cdot 3^{1/4} n^{1/4} +\mathrm{O}(1) \end{aligned}$$

where we used Proposition 3.7 in the second inequality. $\square$

## Convergence to the Wulff Shape

In this section, we use the lower bound (69) on the maximal radius $r_{M_n}$ associated to each minimizer $M_n$ of (2) to study the convergence of minimizers to the hexagonal asymptotic shape as the number *n* of points tends to infinity.

To this end, we recall from the introduction that *W* is the regular hexagon defined as the convex hull of the vectors

$$\begin{aligned} \left\{ \pm \frac{1}{\sqrt{3}}{\varvec{t}}_1,\,\pm \frac{1}{\sqrt{3}}{\varvec{t}}_2,\,\pm \frac{1}{\sqrt{3}}{\varvec{t}}_3\right\} , \end{aligned}$$

where $\mathbf{t_i}$ are defined in Definition 3.2 for $i=1,2,3$. Furthermore, in the following $\mu$ will denote the measure

$$\begin{aligned} \mu :=\frac{2}{\sqrt{3}}\chi _W, \end{aligned}$$

where $\chi _{W}$ is the characteristic function of *W*. We recall that by $\Vert \cdot \Vert$ we denote the total variation norm and by $\Vert \cdot \Vert _\mathrm{F}$ the flat norm defined by

72 $$\begin{aligned} \Vert \mu \Vert _\mathrm{F}:=\sup \left\{ \int _{{\mathbb {R}}^2}\varphi \,d\mu :\,\varphi \text { is Lipschitz with }\Vert \varphi \Vert _{W^{1,\infty }({\mathbb {R}}^2)}\le 1\right\} \end{aligned}$$

for every $\mu \in M_b({\mathbb {R}}^2)$ (see Whitney 1957).

### Proof of Theorem 1.2

In this subsection, we prove Theorem 1.2.

*Step 1* We start by considering

73 $$\begin{aligned} K_n:=\frac{{\left\lceil {\alpha _n}\right\rceil }}{6n^{3/4}}\sqrt{{\left\lceil {\alpha _n}\right\rceil }^2-(\alpha _n)^2}, \end{aligned}$$

where $\alpha _n:=\sqrt{12n-3}$, see (70). In view of the definition of $H_{r_{M_n}}$, we observe that

74 $$\begin{aligned} |M_n\setminus H_{r_{M_n}}|&=n-(1+3\,(r_{M_n})^2+3\,r_{M_n})\nonumber \\&\nonumber \le n\,-\,1\,-\,3\left( \frac{{\left\lceil {\alpha _n}\right\rceil }}{6}-2-\frac{1}{6}\sqrt{{\left\lceil {\alpha _n}\right\rceil }^2-(\alpha _n)^2+33}\right) ^2\nonumber \\&\quad -\,3\left( \frac{{\left\lceil {\alpha _n}\right\rceil }}{6}-2-\frac{1}{6}\sqrt{{\left\lceil {\alpha _n}\right\rceil }^2-(\alpha _n)^2+33}\right) \nonumber \\&= n-\frac{{\left\lceil {\alpha _n}\right\rceil }^2}{12}+\frac{{\left\lceil {\alpha _n}\right\rceil }}{6}\sqrt{{\left\lceil {\alpha _n}\right\rceil }^2-(\alpha _n)^2}+\mathrm{o}(n^{3/4})\nonumber \\&= \frac{{\left\lceil {\alpha _n}\right\rceil }}{6}\sqrt{{\left\lceil {\alpha _n}\right\rceil }^2-(\alpha _n)^2}+\mathrm{o}(n^{3/4}) \end{aligned}$$

where we used Proposition 3.7 in the inequality. Therefore, by (73) and (74) we obtain estimate (24), i.e.,

$$\begin{aligned} |M_n\setminus H_{r_{M_n}}|\le K_n n^{3/4}+\mathrm{o}(n^{3/4}). \end{aligned}$$

Furthermore, since

$$\begin{aligned} \left\| \mu _{M_n}-\mu _{H_{r_{M_n}}}\right\| =\frac{\left| M_n\triangle H_{r_{M_n}}\right| }{n} \end{aligned}$$

and $H_{r_{M_n}}\subset M_n$, by (24) we also obtain that

75 $$\begin{aligned} \left\| \mu _{M_n}-\mu _{H_{r_{M_n}}}\right\| \le K_n n^{-1/4}+\mathrm{o}(n^{-1/4}). \end{aligned}$$

We now define

$$\begin{aligned} d_n:=1+3\,r_{M_n}+3\left( r_{M_n}\right) ^2 \end{aligned}$$

and consider the empirical measure $\mu _{D_{d_n}}$ associated to the daisy $D_{d_n}$. For every point $x_i\in D_{d_n}$, we denote by $Z_i$ the Voronoi cell in ${\mathcal {L}}_t$ related to $x_i$ that is the regular hexagon centered in $x_i$ with side $1/\sqrt{3}$ and edges orthogonal to the three lattice directions. Furthermore, let $Z_i^n:=\{x/\sqrt{n}\,:\,x\in Z_i\}$. We observe that

76 $$\begin{aligned} \Big \Vert \frac{x_i}{\sqrt{n}}-x\Big \Vert _{L^{\infty }(Z_i^n)}\le \frac{1}{\sqrt{3n}}, \end{aligned}$$

and

77 $$\begin{aligned} {\mathcal {L}}^2\left( \left( \bigcup _{i=1}^{d_n} Z_i^n\right) \Delta W\right) = \frac{\sqrt{3}}{2}K_n n^{-1/4}. \end{aligned}$$

For every $\varphi \in W^{1,\infty }({\mathbb {R}}^2)$, we obtain that

78 $$\begin{aligned}&\Big |\int _{{\mathbb {R}}^2}\varphi \,d\mu _{D_n}-\int _{{\mathbb {R}}^2}\varphi \,d\mu \Big |=\Big |\frac{1}{n}\sum _{i=1}^{d_n} \varphi \Big (\frac{x_i}{\sqrt{n}}\Big )-\frac{2}{\sqrt{3}}\int _{W}\varphi \,dx\Big |\nonumber \\&\qquad =\frac{2}{\sqrt{3}}\Big |\sum _{i=1}^{d_n} \varphi \Big (\frac{x_i}{\sqrt{n}}\Big ){\mathcal {L}}^2(Z_i^n)-\int _{W}\varphi \,dx\Big |\nonumber \\&\qquad \le \frac{2}{\sqrt{3}}\Big |\sum _{i=1}^{d_n} \int _{Z_i^n}\Big (\varphi \Big (\frac{x_i}{\sqrt{n}}\Big )-\varphi (x)\Big )\,dx\Big |+\frac{2}{\sqrt{3}}\Vert \varphi \Vert _{L^{\infty }({\mathbb {R}}^2)}{\mathcal {L}}^2\left( \left( \bigcup _{i=1}^{d_n} Z_i^n\right) \Delta W\right) \nonumber \\&\qquad \le \frac{2}{\sqrt{3}}\Vert \nabla \varphi \Vert _{L^{\infty }({\mathbb {R}}^2;{\mathbb {R}}^2)}\sum _{i=1}^{d_n} \int _{Z_i^n}\Big |\frac{x_i}{\sqrt{n}}-x\Big |\,dx +\frac{2}{\sqrt{3}}\Vert \varphi \Vert _{L^{\infty }({\mathbb {R}}^2)}{\mathcal {L}}^2\left( \left( \displaystyle \bigcup _{i=1}^{d_n} Z_i^n\right) \Delta W\right) \nonumber \\&\qquad \le \frac{2}{3\sqrt{n}}\Vert \nabla \varphi \Vert _{L^{\infty }({\mathbb {R}}^2;{\mathbb {R}}^2)}{\mathcal {L}}^2\left( \bigcup _{i=1}^{d_n}Z_i^n\right) +\frac{2}{\sqrt{3}}\Vert \varphi \Vert _{L^{\infty }({\mathbb {R}}^2)}{\mathcal {L}}^2\left( \left( \displaystyle \bigcup _{i=1}^{d_n} Z_i^n\right) \Delta W\right) \nonumber \\&\qquad \le \frac{2}{3\sqrt{n}}\Vert \nabla \varphi \Vert _{L^{\infty }({\mathbb {R}}^2;{\mathbb {R}}^2)}\displaystyle \frac{\displaystyle {\mathcal {L}}^2({\hat{H}}_{r_{M_n}+1})}{n}+\frac{2}{\sqrt{3}}\Vert \varphi \Vert _{L^{\infty }({\mathbb {R}}^2)}{\mathcal {L}}^2\left( \left( \bigcup _{i=1}^{d_n} Z_i^n\right) \Delta W\right) \nonumber \\&\qquad \le \Vert \varphi \Vert _{W^{1,\infty }({\mathbb {R}}^2)}\mathrm{O}(n^{-1/2}) + \Vert \varphi \Vert _{L^{\infty }({\mathbb {R}}^2)}K_n n^{-1/4}, \end{aligned}$$

where we used (76) and (77) in the third and the last inequality, respectively.

By combining (75) with (78), we obtain that

79 $$\begin{aligned} \mu _{M'_n}\rightharpoonup ^*\mu \quad \text {weakly* in }M_b({\mathbb {R}}^2), \end{aligned}$$

and

80 $$\begin{aligned} \left\| \mu _{M'_n}-\mu \right\| _\mathrm{F}\le 2 K_n n^{-1/4}+\mathrm{o}(n^{-1/4}), \end{aligned}$$

where $M'_n:=M_n-q_n$, with $q_n\in {\mathcal {L}}_t$ such that $H_{r_{M_n}}=D_{1+3r_{M_n}+3r_{M_n}^2}+q_n$.

*Step 2* Assertions (15)–(18) directly follow from (24), (75), and (80) since by (70) and (73) a direct computation shows that

81 $$\begin{aligned} K_n&=\frac{{\left\lceil {\alpha _n}\right\rceil }}{6n^{3/4}}\sqrt{{\left\lceil {\alpha _n}\right\rceil }^2-(\alpha _n)^2}\nonumber \\&=\frac{2}{3^{1/4}}\sqrt{\left\lceil \sqrt{12n-3}\,\right\rceil -\sqrt{12n-3}}+\mathrm{o}(1)\nonumber \\&=K_t\sqrt{\left\lceil \sqrt{12n-3}\,\right\rceil -\sqrt{12n-3}}+\mathrm{o}(1) \end{aligned}$$

We notice here that Theorem 1.2 implies in particular the convergence (up to translations) of the empirical measures associated with the minimizers to the measure $\mu$ not only with respect to the weak$*$-converge of measures, but also with respect to the flat norm [see (72)].

We remark that an alternative approach to the one adopted in Theorem 1.2 is that of defining a unique *n*-configurational Wulff shape $W_n$ for all the minimizer with *n* atoms. For example, we could define

$$\begin{aligned} W_n:={\hat{W}}_n\cap {\mathcal {L}}_t, \end{aligned}$$

where ${\hat{W}}_n$ is the hexagon with side $p_n/6$ and center $x_{\tau (1)}$. We remark that the $\mathrm{O}(n^{1/4})$ estimate on the Hausdorff distance and the $\mathrm{O}(n^{3/4})$-law still hold true by replacing the maximal hexagon $H_{r_{M_n}}$ with $W_n$.

More precisely, by Proposition 3.7 we have that

82 $$\begin{aligned} d_{{\mathcal {H}}}(W_n,H_{r_{M_n}})\le 6\Big |\frac{p_n}{6}-r\Big |\le \sqrt{{\left\lceil {\alpha _n}\right\rceil }^2-(\alpha _n)^2}+\mathrm{O}(1) \end{aligned}$$

and that

83 $$\begin{aligned} |W_n \setminus H_{r_{M_n}}|&\le \Big |3\Big (\left\lfloor \frac{p_n}{6} \right\rfloor \Big )^2+3\Big (\left\lfloor \frac{p_n}{6} \right\rfloor \Big )-3\left( r_{M_n}\right) ^2-3\,r_{M_n}\Big |\nonumber \\&=3\Big (\left\lfloor \frac{p_n}{6} \right\rfloor +r_{M_n}+1\Big )\Big |\left\lfloor \frac{p_n}{6} \right\rfloor -r_{M_n}\Big |\nonumber \\&\le \frac{p_n}{6}\sqrt{{\left\lceil {\alpha _n}\right\rceil }^2-(\alpha _n)^2}+\mathrm{o}(n^{3/4})\nonumber \\&=\frac{{\left\lceil {\alpha _n}\right\rceil }}{6}\sqrt{{\left\lceil {\alpha _n}\right\rceil }^2-(\alpha _n)^2}+\mathrm{o}(n^{3/4}) \end{aligned}$$

for every minimizer $M_n$. Therefore, we obtain that

$$\begin{aligned} d_{{\mathcal {H}}}(M'_n, W_n)\le \mathrm{O}(n^{1/4}) \end{aligned}$$

by (20) and (82), and

84 $$\begin{aligned} \left| M'_n \triangle W_n\right| \le \mathrm{O}(n^{3/4}) \end{aligned}$$

by (24) and (84), with $M'_n:=M_n-q_n$ where $q_n\in {\mathcal {L}}_t$ are chosen in such a way that

$$\begin{aligned} H_{r_{M_n}}=D_{1+3r_{M_n}+3r_{M_n}^2}+q_n. \end{aligned}$$

Furthermore, from (84) it follows that

$$\begin{aligned} \left\| \mu _{M'_n}-\mu _{W_n}\right\| = \frac{\left| M'_n \triangle W_n\right| }{n}\le \mathrm{O}(n^{-1/4}). \end{aligned}$$

### Proof of Theorem 1.4

In this subsection, we prove that the estimates (15)–(17) are sharp.

*Step 1* In this step, we show that there exists a sequence of minimizers ${\overline{M}}_n$ such that, denoting by $H_{r_{\,{\overline{M}}_n}}$ their maximal hexagons,

85 $$\begin{aligned} |{\overline{M}}_n\setminus H_{r_{\,{\overline{M}}_n}}|=K_n n^{3/4}+\mathrm{o}(n^{3/4}). \end{aligned}$$

**Fig. 6:**
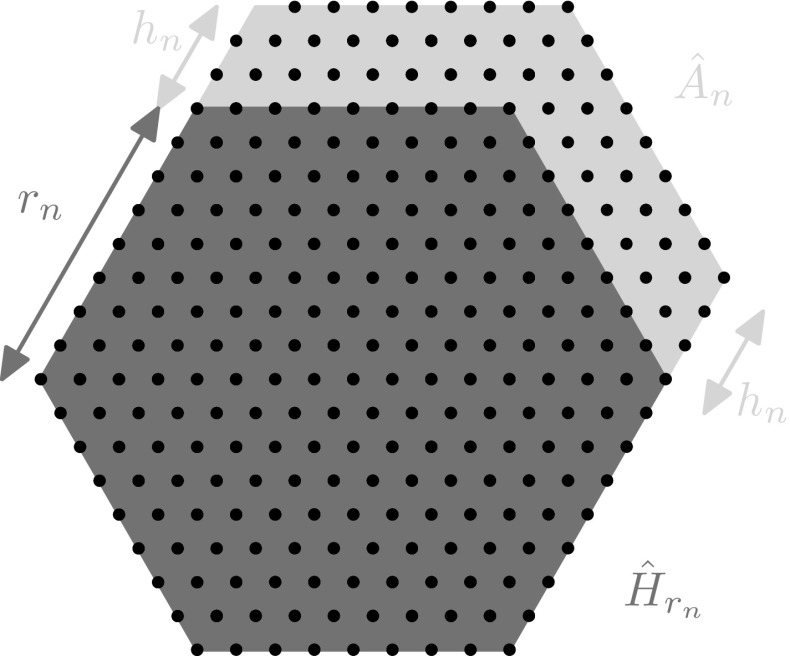
The form of a minimizer ${\overline{M}}_n$ constructed in the proof of Theorem 1.4 is shown. The configuration ${\overline{M}}_n$ is contained in the union of the hexagon ${\hat{H}}_{r_n}$ drawn in the darkest color (*blue*) and the region ${\hat{A}}_n$ constructed on two of its sides drawn in the lightest color (*yellow*) (Color figure online)

We will explicitly construct the minimizers ${\overline{M}}_n$. To this end, we denote by ${\hat{H}}_{r_n}$ the closure of the regular hexagon in $\mathbb {R}^2$ with center in $x_{\tau (1)}$ and side $r_n$ defined by

$$\begin{aligned} r_n:={\left\lceil {\frac{{\left\lceil {\alpha _n}\right\rceil }}{6}-\frac{1}{6}\sqrt{{\left\lceil {\alpha _n}\right\rceil }^2-(\alpha _n)^2}}\right\rceil }, \end{aligned}$$

and we introduce $H_{r_n}:={\hat{H}}_{r_n}\cap {\mathcal {L}}_t$. Furthermore, we define

$$\begin{aligned} h_n:=\frac{p_n}{2}-3r_n \end{aligned}$$

and we consider the region

$$\begin{aligned} {\hat{A}}_n:= \{x+h_n{\varvec{t}}_2\ : \ x\in {\hat{H}}_{r_n}\}\setminus {\hat{H}}_{r_n} \end{aligned}$$

that consists of two parallelograms of height $h_n$ constructed on two consecutive sides of $H_{r_n}$ (see Fig. 6).

Let $c:=|({\hat{H}}_{r_n}\cup {\hat{A}}_n)\cap {\mathcal {L}}_t|$. We denote by $C_c$ the configuration defined by

$$\begin{aligned} C_c:=\left( {\hat{H}}_{r_n}\cup {\hat{A}}_n\right) \cap {\mathcal {L}}_t \end{aligned}$$

and we observe that, by construction, the perimeter of $C_c$ satisfies

86 $$\begin{aligned} P(C_c)=p_n. \end{aligned}$$

We subdivide the remaining proof of the claim into two substeps.

*Substep 1.1.* We claim that for every *n* big enough there exists a minimizer ${\overline{M}}_n$ such that

$$\begin{aligned} H_{r_n}\subseteq {\overline{M}}_n \subseteq C_c \end{aligned}$$

and $$|C_c\setminus {\overline{M}}_n|\le 2r_n-1$$.

We begin by observing that

87 $$\begin{aligned} c:=|C_c|&=|H_{r_n}|+(2r_n+1)h_n\nonumber \\&=1+3r_n^2+3r_n+\Big (r_n+\frac{1}{2}\Big )(p_n-6r_n)\nonumber \\&=-3r_n^2+p_nr_n+1+\frac{p_n}{2}. \end{aligned}$$

Then, a direct computation shows that

88 $$\begin{aligned} 3s^2-p_n s-1-\frac{p_n}{2}\ge 0 \end{aligned}$$

for every $s\in \left[ \displaystyle \frac{{\left\lceil {\alpha _n}\right\rceil }}{6}\,-3\,-\frac{1}{6}\sqrt{{\left\lceil {\alpha _n}\right\rceil }^2+3} , \frac{{\left\lceil {\alpha _n}\right\rceil }}{6}\,-3\,+\frac{1}{6}\sqrt{{\left\lceil {\alpha _n}\right\rceil }^2+3} \right]$, and, for *n* big enough,

89 $$\begin{aligned} 3s^2+(2-p_n)s-2-\frac{p_n}{2}+n\ge 0 \end{aligned}$$

for every $s\in {\mathbb {R}}$. In particular, (88) and (89) hold for $s=r_n$ and for *n* sufficiently large, yielding

90 $$\begin{aligned} 0\le c-n\le 2r_n-1. \end{aligned}$$

We now observe that by the definition of $C_c$ it is possible to remove up to $2r_n-1$ points from $C_c\setminus H_{r_n}$ without changing the perimeter of the configuration. In view of (90), we construct ${\overline{M}}_n$ by removing in such a way $c-n$ points from $C_c$. It follows from (86) that $P({\overline{M}}_n)=p_n$ and hence, the claim holds true.

*Substep 1.2.* Let ${\overline{M}}_n$ be the sequence of ground states constructed in the previous substep. In view of (90), and of the definition of $\alpha _n$ and $p_n$, there holds

91 $$\begin{aligned} |C_n\setminus H_{r_n}|&=(2r_n+1)h_n\nonumber \\&=-6(r_n)^2-3r_n+p_nr_n+1+\frac{p_n}{2}\nonumber \\&=\frac{{\left\lceil {\alpha _n}\right\rceil }}{6}\sqrt{{\left\lceil {\alpha _n}\right\rceil }^2-(\alpha _n)^2}+\mathrm{o}(n^{3/4}). \end{aligned}$$

Moreover, by the definition of ${\overline{M}}_n$ we have that

92 $$\begin{aligned} |C_n\setminus {\overline{M}}_n|\le 2r_n-1=\mathrm{O}(n^{1/2})=\mathrm{o}(n^{3/4}). \end{aligned}$$

The thesis follows from combining (91) and (92) since $H_{r_n}$ is by construction the maximal hexagon of ${\overline{M}}_n$.

*Step 2* In this last step, we remark that

$$\begin{aligned} \limsup _{n\rightarrow +\infty }K_n=K_t\limsup _{n\rightarrow +\infty }\sqrt{\left\lceil \sqrt{12n-3}\,\right\rceil -\sqrt{12n-3}}\le K_t,\end{aligned}$$

and that for those $n_j\in {\mathbb {N}}$ of the form $n_j=2+3j+3j^2$ there holds

93 $$\begin{aligned} K_{n_j}\rightarrow \frac{2}{3^{1/4}}=:K_t \end{aligned}$$

as $$j\rightarrow +\infty $$.

In fact, we have that

$$\begin{aligned} \sqrt{12n_j-3}&=\sqrt{12(1+3j+3j^2)+9}\nonumber \\&=(6j+3)\sqrt{1+\frac{12}{(6j+3)^2}}\\&=6j+3+\frac{12}{(6j+3)\left[ \,1+\sqrt{1+\displaystyle \frac{12}{(6j+3)^2}}\,\right] }, \end{aligned}$$

which in turn yields

$$\begin{aligned} \left\lceil \sqrt{12 n_j -3}\,\right\rceil -\sqrt{12 n_j -3}=1-\frac{12}{(6j+3)\left[ \,1+\sqrt{1+\displaystyle \frac{12}{(6j+3)^2}}\,\right] }\rightarrow 1\end{aligned}$$

as $$j\rightarrow +\infty $$.$$\square $$

It is remarkable that the leading terms in the estimates (24), (75), and (80) established in Step 1 of Theorem 1.2 are optimal for every $n\in \mathbb {N}$ as it follows from Step 1 of the proof of Theorem 1.4.

Finally, we notice that the bounded quantities $K_n$ defined in (73) are 0 for every $n\in {\mathbb {N}}$ that can be written as $n=1+3k+3k^2$ for some $k\in {\mathbb {N}}$. This reflects the fact that for those *n* the daisy $D_n$ is the unique minimizer, whose maximal hexagon $H_{r_{D_n}}$ is the daisy itself. Therefore, Theorem 1.4 also entails that, by adding a point to every EIP (2) with $n=1+3i+3i^2$ for some $i\in \mathbb {N}$, we pass not only from a problem characterized by uniqueness of solutions to a problem with nonuniqueness, but also from a situation of zero deviation of the minimizer from its maximal hexagon to the situation in which minimizers include one that attains the maximal deviation.
